# Integrative modeling of lncRNA-chromatin interaction maps reveals diverse mechanisms of nuclear retention

**DOI:** 10.1186/s12864-023-09498-9

**Published:** 2023-07-13

**Authors:** Shayan Tabe-Bordbar, Saurabh Sinha

**Affiliations:** 1 Department of Computer Science, University of Illinois at Urbana-Champaign, Urbana, IL USA; 2 Department of Biomedical Engineering, Georgia Institute of Technology, UAW 3108, 313 Ferst Drive NW, Atlanta, GA 30332 USA

**Keywords:** Long non-coding RNA, Nuclear localization, Chromatin interaction, DNA Damage Response, R-loop, Machine learning

## Abstract

**Background:**

Many long non-coding RNAs, known to be involved in transcriptional regulation, are enriched in the nucleus and interact with chromatin. However, their mechanisms of chromatin interaction and the served cellular functions are poorly understood. We sought to characterize the mechanisms of lncRNA nuclear retention by systematically mapping the sequence and chromatin features that distinguish lncRNA-interacting genomic segments.

**Results:**

We found DNA 5-mer frequencies to be predictive of chromatin interactions for all lncRNAs, suggesting sequence-specificity as a global theme in the interactome. Sequence features representing protein-DNA and protein-RNA binding motifs revealed potential mechanisms for specific lncRNAs. Complementary to these global themes, transcription-related features and DNA-RNA triplex formation potential were noted to be highly predictive for two mutually exclusive sets of lncRNAs. DNA methylation was also noted to be a significant predictor, but only when combined with other epigenomic features.

**Conclusions:**

Taken together, our statistical findings suggest that a group of lncRNAs interacts with transcriptionally inactive chromatin through triplex formation, whereas another group interacts with transcriptionally active regions and is involved in DNA Damage Response (DDR) through formation of R-loops. Curiously, we observed a strong pattern of enrichment of 5-mers in four potentially interacting entities: lncRNA-bound DNA tiles, lncRNAs, miRNA seed sequences, and repeat elements. This finding points to a broad sequence-based network of interactions that may underlie regulation of fundamental cellular functions. Overall, this study reveals diverse sequence and chromatin features related to lncRNA-chromatin interactions, suggesting potential mechanisms of nuclear retention and regulatory function.

**Supplementary Information:**

The online version contains supplementary material available at 10.1186/s12864-023-09498-9.

## Background

Long non-coding RNAs (lncRNAs) are 200 bp or longer transcripts lacking a substantial open reading frame (ORF), that have been shown to be functionally significant in many different contexts [1]. Cell-type specificity and gene regulatory functions of lncRNAs point us to their potential significance in organismal complexity [2] and disease [3]. Many lncRNAs are preferentially retained in the nucleus where they perform a variety of regulatory functions, and the elucidation of such functions is an important frontier of research today [4].

Mechanisms underlying nuclear enrichment of lncRNAs are not well understood and anecdotal evidence suggests these are diverse and combinatorial [5]. A plausible mechanism is the interaction of lncRNAs with nuclear proteins and/or chromatin. This is supported by reports of direct lncRNA-DNA interactions via triplex formation [6] or through R-loops [7], as well as indirect interactions mediated by RNA-binding proteins (RBPs) or chromatin modifiers [8]. Emerging technologies have been used to catalog RNA-chromatin interactions genome-wide [9] and have revealed individual lncRNAs to bind to or localize at specific genomic regions; such specific localization has in turn been linked to the RNA’s biological functions [10, 11]. These findings motivate the question: what characterizes the genomic segments that a specific lncRNA is found to localize at? Sequence patterns such as those found in Alu repeat elements [12] and generic sequence properties such as high GA content [13] have been reported as characterizing the bound regions. However, a thorough examination of the sequence, structural and epigenomic signatures distinguishing chromatin interaction sites for individual lncRNAs has not been undertaken.

**Fig. 1:**
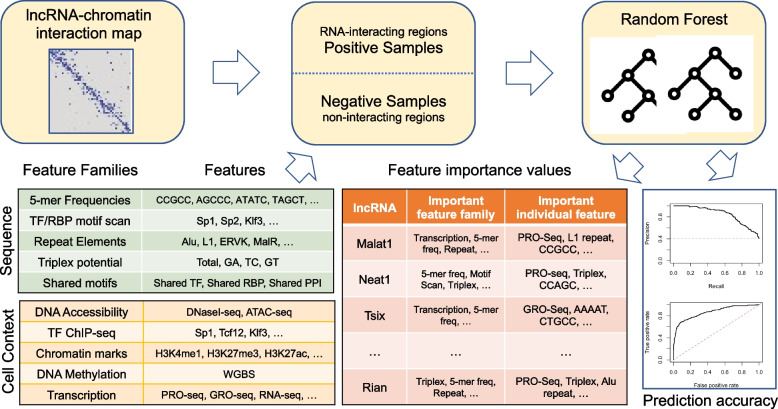
Study overview. This study analyzes RNA-chromatin interaction maps (top left) obtained from publicly available RADICL-Seq and GRID-Seq experiments conducted on mouse embryonic stem cells. We segmented the mouse genome into non-overlapping 1 kb tiles, identified 28 lncRNAs that interact with at least 1000 unique tiles, and focused on these lncRNAs. To learn about the distinguishing characteristics of lncRNA-interacting DNA tiles, we set up a classification task aimed to discern such tiles from the ones in their proximity that do not interact with a lncRNA (top middle). We employed Random Forest (RF) classifiers using families of features characterizing the tiles’ sequence and cellular context (bottom left). We ranked features and feature families based on their average contribution to the predictive power of the classifier (bottom middle). Feature importance analysis focused on lncRNAs for which cross-validation revealed predictive accuracy (bottom right) above a pre-set threshold

Here, we sought to identify properties related to sequence or chromatin state that distinguish the genomic segments found to interact with specific lncRNAs (Fig. 1). We expected statistically identified properties to provide support for suggested as well as novel mechanisms of lncRNA-chromatin interaction, ultimately shedding light on lncRNA nuclear retention and functions. We analyzed RNA-chromatin interaction data from mouse embryonic stem cells (mESC), obtained using GRID-seq [14] and RADICL-seq [15] technologies and representing dozens of lncRNAs. We trained machine learning models to distinguish the lncRNA-interacting DNA regions from carefully selected non-interacting regions for each lncRNA. This classification task was defined in a way that forces models to focus on distinguishing features other than distance to the lncRNA gene, the predominant factor influencing chromatin localization. The models leverage information from lncRNA and DNA sequence as well as a broad range of experimental measurements from mESC. Sequence information includes counts of all 5-mers as well as computationally predicted binding sites of transcription factors (TFs) or RBPs and presence of repeat elements. Experimental measurements used by the models include binding profiles of DNA-binding proteins, chromatin marks, transcription measurements as well as methylation and accessibility profiles from mESC. We systematically examined the contribution of each of thousands of features, as well as families of features, to the predictive ability of the models. Our analysis was carefully designed to account for the extensive correlations known to be present among different epigenomic features and the partially causal relationship between sequence and epigenomic states.

The studied lncRNAs fell into two broad categories: for one category, transcription-related features are highly predictive of chromatin interactions, while for the other the triplex-formation potential between the RNA and DNA is a strong predictor. DNA methylation-related information, when combined with other epigenomic features, was also noted to be a significant marker of lncRNA localization. Sequence footprints were highly informative for the classification task, with the most useful features representing protein-DNA and protein-RNA binding, triplex formation, and specific repeat elements. Curiously, we observed a persistent association between microRNA seed sequences and lncRNA-chromatin interaction, suggesting that mechanisms similar to “competing endogenous RNA” [16] may be involved in nuclear retention as well. Finally, we noted several predictive features used by the models as being related to DNA Damage Response, supporting the broad role played by non-coding RNAs in this process [10, 17]. In summary, our work presents a systematic and extensive catalog of potential mechanisms underlying chromatin localization of a diverse compendium of lncRNAs and highlights broader patterns of mechanisms shared across lncRNAs.

## Results

### The lncRNA-chromatin interactome: dominance of cis interactions

**Fig. 2:**
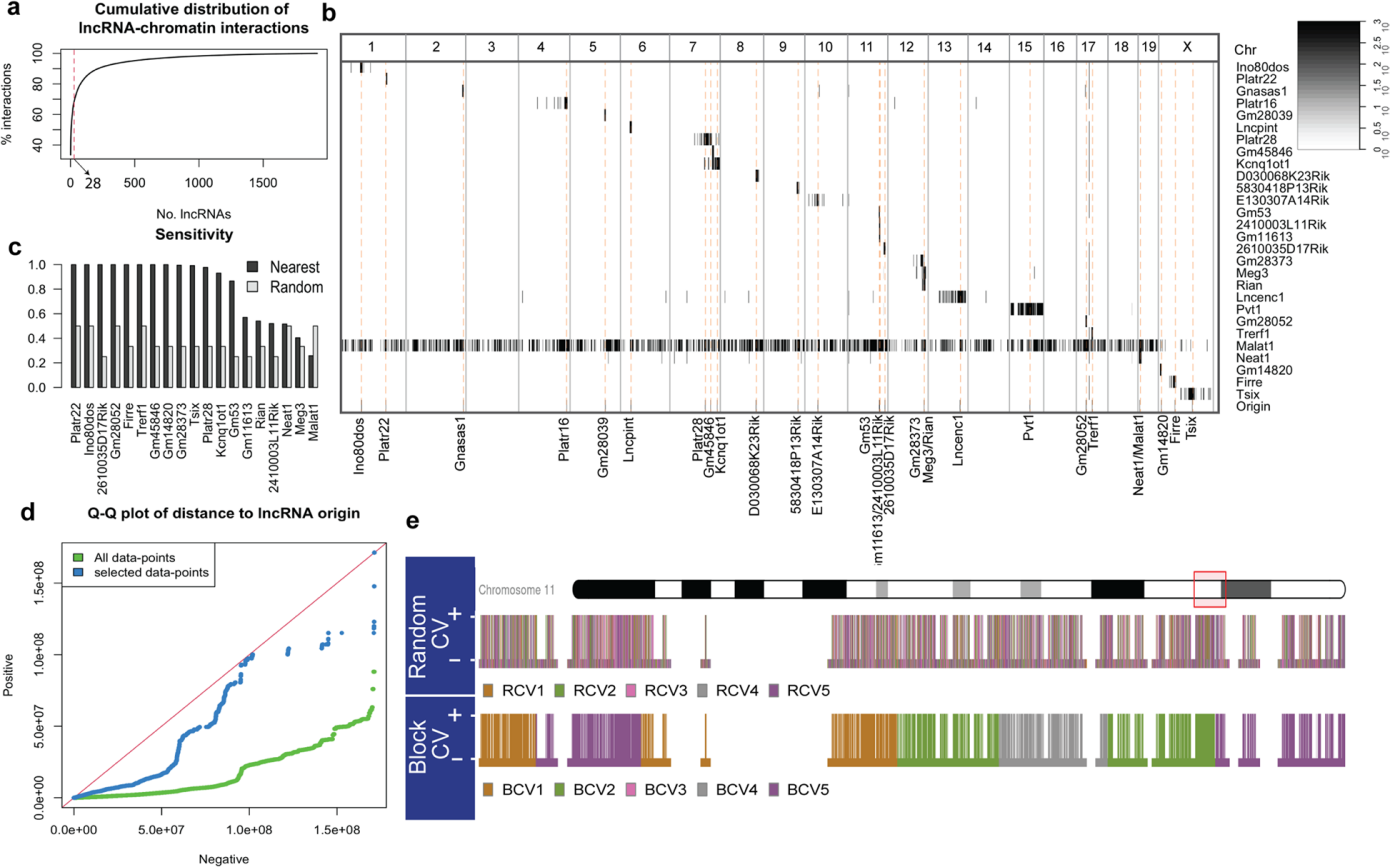
**a** Cumulative distribution of unique interactions. For any x (number of lncRNAs), the corresponding y indicates the percentage of interactions spanned by the top x lncRNAs (sorted by number of interactions). Dashed red line indicates the number of lncRNAs considered in this study. The selected 28 lncRNAs form about 68% of all interactions. **b** Interaction frequency matrix, representing the number of interactions formed by each lncRNA (rows) with all 1 Mb-DNA regions (columns). Bottom row (and dashed orange line) indicates the origin of biosynthesis (gene) for the considered lncRNAs. **c** Performance (i.e., sensitivity) comparison of random (“Random”) and distance-based (“Nearest”) classifiers that identify, for each lncRNA-interacting DNA tile, the lncRNA it interacts with, among all lncRNAs originating from the same chromosome. Distance-based classifier assigns each lncRNA-bound DNA tile to the lncRNA whose gene is located closest to that tile, while the random classifier makes the assignment by selecting one at random among all the cis-located lncRNAs (i.e., lncRNAs originating on the same chromosome as the DNA tile). **d** Each Q-Q plot compares lncRNA-bound (“Positive”) and unbound (“Negative”) DNA tiles in terms of their distance (in bp) from their interacting lncRNA’s origin of biosynthesis. The two plots represent all data-points (green) or the subset of data-points provided to the classiffiers (blue), respectively. **e** Visual comparison of Random and Block cross-validation schemes for Malat1-chromatin interactions, focusing on a region of chromosome 11. Vertical lines represent tiles subjected to classification. The height of vertical lines indicates their label (tall for positive, short for negative) and their color specifies the partition to which they belong in a five-fold cross validation setup

We collected data from GRID-Seq [14] and RADICL-Seq [15] experiments conducted in mouse Embryonic Stem Cells (mESC). LncRNA-chromatin interactions were extracted from both data sets to account for their complementary strengths and biases. These data included 751,893 interactions between lncRNAs and distinct 1 kb-long DNA tiles, the majority of which are mediated by relatively few lncRNAs (Fig. 2a). We focused on the 28 lncRNAs that interact with at least 1000 unique 1 kb DNA tiles and together mediate 68% of all interactions, involving 180,220 unique tiles whose genomic distributions are shown in Fig. 2b. We noted that Malat1 binds to far greater number of DNA tiles compared to other lncRNAs – 110,183 unique 1-kb tiles, more than six times as many tiles as any other lncRNA. Furthermore, Malat1 is the only lncRNA among the 28 with “trans” interactions, i.e., interactions with DNA regions not located on the same chromosome as the lncRNA gene. In cases where a chromosome harbors more than one of the considered lncRNAs (e.g., Platr28, Gm45846, and Kcnq1ot1 located on chromosome 7), we observed each lncRNA to interact with DNA regions closer to its gene. In fact, within this data set, if a DNA tile is known to be bound by some lncRNA in cis, the interacting lncRNA can be identified accurately merely based on distance to lncRNA genes (Fig. 2c). This striking predominance of proximal interactions might be explained by the generally low expression of lncRNAs and the resulting concentration gradient in their cellular microenvironment [18].

### Machine Learning framework to identify mechanisms of lncRNA-chromatin interactions

We found above that lncRNAs generally bind DNA in the vicinity of their respective genes. However, not all neighboring DNA tiles are bound by the lncRNA. This raises the possibility that factors besides distance affect the interaction. We hypothesized that sequence characteristics of the DNA tile (“sequence features”) or features obtained from various experimental measurements in mESCs (“cell-context features”) might be predictive of lncRNA-chromatin interactions. Identifying distinctive characteristics of adjacent but differentially bound DNA tiles might shed light on the mechanisms involved in lncRNA-chromatin interaction and provide clues about the functional role of such interactions in cellular processes.

#### Classification, features and feature importance

Features used to describe each DNA tile (Table 1) fall into two main categories – sequence features and cell-context features – with each category comprising five families of features, and each family comprising between one and 701 features (see Methods for details). Most features capture information about the DNA tiles alone, but some capture information specific to a lncRNA-tile pair. We used the full set of 1613 features to train a Random Forest (RF) classifier for each lncRNA. The classifier distinguishes the DNA tiles interacting with a specific lncRNA from non-interacting ones (details below). We evaluated its predictive ability on unseen test sets of DNA tiles (details below). We assessed the importance of each feature family by comparing the predictive performance of models that differ only in the inclusion or exclusion of that family (Methods). Finally, we cataloged the importance of individual features to the predictive ability of the classifier for each lncRNA (Fig. 1 and Methods).

**Table 1:** Features and feature families, belonging to sequence category or cell-context category, used to describe each DNA tile

	**Family**	**Features**
Sequence	k-mer frequency	512 features, each representing the count of a 5-mer (and its reverse complement) in the DNA tile.
	Motif scan	701 features obtained by counting matches to TF motifs (623) or RBP motifs (78) in a DNA tile or its putative transcript respectively. TFs and RBPs are known to mediate lncRNA-chromatin interactions [8] and the “motif scan” feature family may capture such factors using their nucleic acid binding preferences.
	Repeat element count	60 features, 59 of which represent the number of times a tile overlaps with one of 59 different repeat elements obtained from RepeatMasker [19]. The last feature in this family, named “repeat-pair”, is the count of distinct repeat elements that overlap with both the DNA tile and lncRNA. Repeat elements have been proposed to play a role in nuclear localization of lncRNAs [12], motivating our use of this family of sequence features.
	Triplex formation potential	Four features quantifying the triplex formation potential as predicted by the Triplexator tool [20], based on the sequence of DNA and lncRNA. LncRNAs have been observed to form triplex structures with double stranded DNA by forming Hoogsteen bonds [6].
	Shared motifs	Three features, viz., “TF-pair”, “RBP-pair”, and “PPI-pair”. Each feature is the count of proteins of a specific class (TFs, RBPs) or of protein complexes that have motif matches to both the DNA tile and the lncRNA sequence. These features quantify the number of potential mediators for a DNA-lncRNA interaction.
Cell Context	DNA accessibility	Two features reflecting DNase-Seq and ATAC-Seq accessibility profiles in mESC.
	DNA methylation	Single feature obtained from a Whole Genome Bisulfite Sequencing (WGBS) assay conducted on mESC.
	Chromatin marks	48 features corresponding to ChIP-Seq profiles of distinct histone modifications in mESC, as such modifications are known markers and/or determinants of genome function. The family includes an additional feature called “chromatin-pair” that quantifies the chromatin state similarity (number of shared chromatin marks) between the lncRNA gene and DNA tile, motivated by reports of similar chromatin state at interacting chromatin domains [21].
	TF ChIP	275 features quantifying the binding of TFs and other DNA-binding proteins to each DNA tile in mESC, as well as a feature that reflects the number of proteins observed to bind both the lncRNA gene and the DNA tile.
	Transcription	Five features describing the nascent and processed transcription levels at the DNA tile, as well as RNA-pol II binding profile.

#### Choice of “positive” and “negative” sets for classification

Positive examples for a specific lncRNA are the DNA tiles interacting with that lncRNA. For negative examples, DNA tiles that do not interact with the lncRNA are selected, but they are strategically positioned near the positive tiles. Essentially, our objective was to choose positive and negative examples that cannot be easily distinguished based on the genomic distance from the lncRNA gene. To accomplish this, we sampled negative tiles in a way that their distribution of distances from the lncRNA gene closely resembled the distribution observed for positive tiles (see Fig. 2d).

#### Model evaluation

To assess the predictive capability of models that make genomic predictions in a single cell type, cross-chromosomal evaluation is a commonly used method. In this version of the "k-fold" Cross-Validation (CV) strategy, the genomic regions on a chromosome are combined to create the test set for a CV "fold" or iteration. This CV strategy is known to provide more realistic estimates of model generalizability [22]. However, our analysis revealed that the majority of the considered lncRNAs interact exclusively with tiles located on a single chromosome, rendering cross-chromosomal CV ineffective for our purposes (as depicted in Fig. 2b). Nonetheless, drawing inspiration from it, we devised a new CV approach called "block CV." In this approach, we divide all the tiles on a chromosome into contiguous blocks, ensuring that tiles within a block remain together as part of either the test set or the training set in any CV fold. We evaluated all the trained models using fivefold block CV, as well as a fivefold random CV where tiles are randomly assigned to the training and test sets regardless of their block identity. An example of both CV strategies is illustrated in Fig. 2e.

### Full models predict chromatin interactions for a majority of lncRNAs

**Fig. 3:**
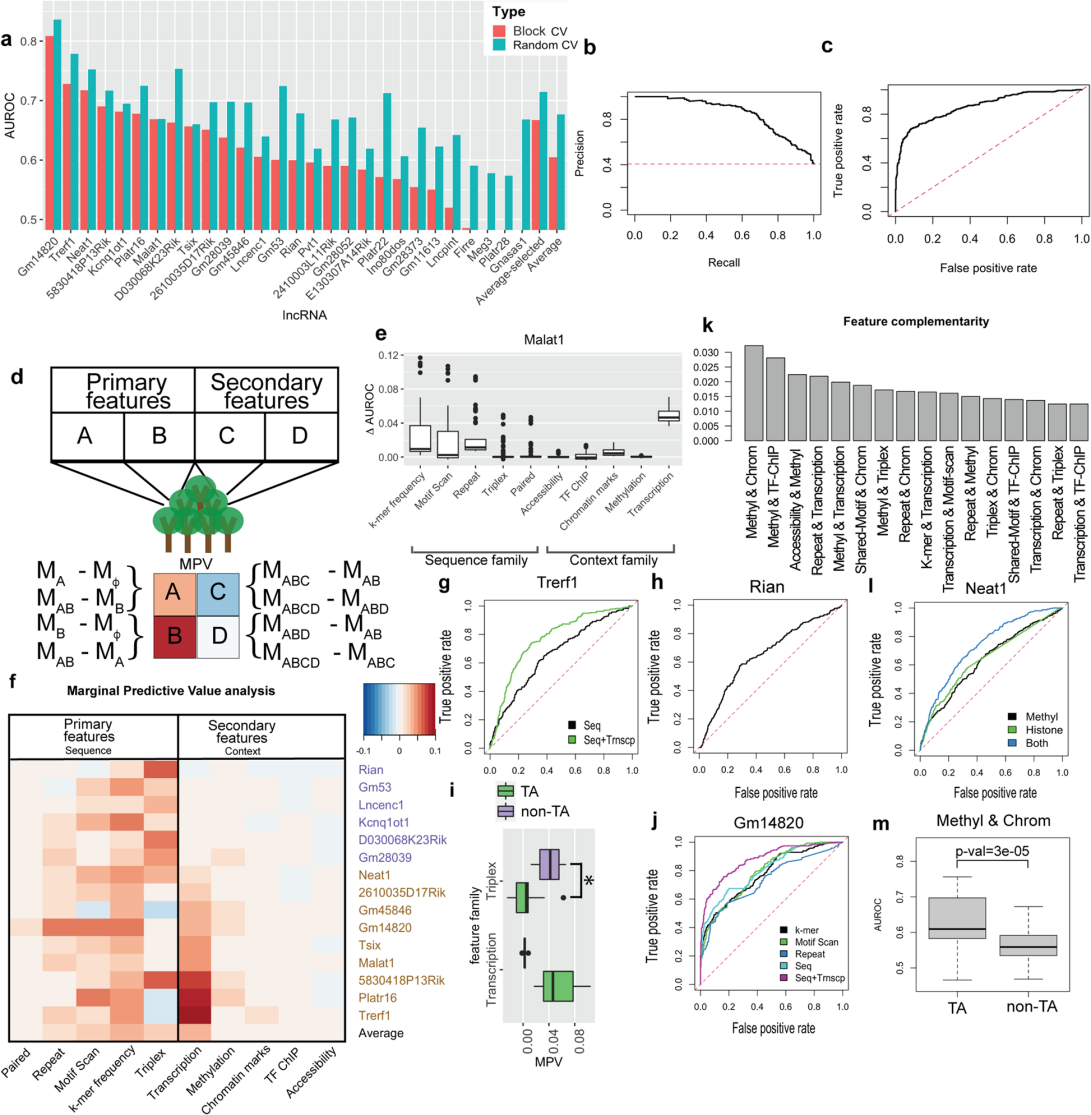
Model performance and feature family analysis. **a** Bar chart illustrates the test performance of the RF classifier using all features, for each lncRNA in random/block cross-validation settings. **b** and **c** illustrate PR and ROC curves respectively, quantifying the test performance of RF models trained to predict Gm14820-chromatin interactions. **d** Schematic representation of the Marginal Predictive Value (MPV) evaluation framwork. The toy example is a scenario with two primary and two secondary families of features. The MPV of a feature family is quantified as the average difference in test performance of pairs of models that have been trained using feature sets that only differ in the inclusion of that feature family (see Methods). **e** Boxplots (median and quartiles) show the contributions of feature families to the prediction of Malat1-chromatin interactions. Each boxplot corresponds to one feature family and depicts the distribution of 16 points representing pairwise differences in performance of models differing only in inclusion of that particular feature family. **f** Heatmap shows MPV of feature familes (columns) for all lncRNAs (rows). Row label colors show lncRNA group (TA and non-TA lncRNAs shown in gold and navy, respectively). **g** Black line shows the test ROC of a classifier that uses sequence features only, whereas the green line shows the test ROC of a classifier that uses the transcription family of features in addition to sequence features to predict Trerf1-chromatin interactions. **h** ROC curve shows the performance of a classifier that uses triplex feature family to predict chromatin interactions for Rian. **i** Boxplots illustrate the MPV for Triplex and Transcription feature families grouped by lncRNA type. * indicates Mann–Whitney *p*-value of 0.01. **j** ROC curves showing performance of models trained to predict Gm14820 chromatin interactions. **k** Average complementarity scores for 16 top-scoring pairs of feature families are shown. Chrom: Chromatin marks; Methyl: Methylation. **l** ROC curves of models trained to predict Neat1-chromatin interactions using DNA methylation alone (black), Histone marks alone (green), or both (blue) as predictors. **m** Boxplots indicate the performance of models trained using methylation and chromatin mark features for all lncRNAs grouped by their type

To assess the overall predictive power of the defined features, we used all feature families together to train an RF model for each lncRNA. Figure 3a shows block and random CV test performance of the trained full model for each lncRNA. Random CV generally provides a more optimistic estimate of performance as compared to block CV. It is notable that models trained for majority of the considered lncRNAs, including Malat1 and Neat1, performed considerably better than a random classifier (expected AUROC = 0.5) in either CV scheme. Gm14820 interactions are particularly well-predicted (block AUROC = 0.81, averaged across the 5 folds) by the model. ROC and PR curves for the prediction of Gm14820-interacting tiles, from one of the block CV partitions, are reported in Fig. 3b and c, respectively. An example of model predictions for this lncRNA is shown in Supplementary Fig. S1. We set block test AUROC of 0.6 (average across five folds) as the predictability threshold and chose to focus on the 15 lncRNAs (of the initially considered 28) that pass this threshold, to better understand the most predictive features.

### Transcription and triplex formation potential are predictive of chromatin interactions of two distinct groups of lncRNAs

Two main mechanisms of lncRNA-chromatin interaction [8] are (1) indirect interactions mediated by chromatin modifiers and proteins with dual DNA/RNA binding capacity, and (2) direct interactions, either through triplex structures formed between single stranded RNA and double stranded DNA, or through R-loop formation. We hypothesized that these and other mechanisms will be reflected in the predictive power of specific feature families used by our classifiers. Cognizant of the inter-dependence and correlation among various features, we devised the following approach to assess the predictive power of each family of features for a given lncRNA. We first train RF models using all possible combinations of feature families: given *n* feature families, we train 2^n^—1 models each including a unique combination of feature families. (A feature family being included means that all features in that family are included in the model.) Next, to identify the predictive ability of a feature family, we compare the CV test AUROC between pairs of models that differ only in inclusion of that feature family. The performance boost gained by the addition of a particular feature family to any baseline model provides us with a measure of predictive value of that family, and we call its average, over all choices of the baseline model, the “Marginal Predictive Value” (MPV) for that feature family. This approach is inspired by a recently developed model interpretation framework, called SHAP [23]. Catav et al. [24] suggest the use of maximum (as opposed to average) gain of performance achieved by addition of a feature, as its marginal contribution. Following their work, we repeated our analyses with Maximal Predictive Value (MxPV, see methods) in place of MPV, and noted the overall trends to remain unchanged. Hence, we only report the results from MPV analysis here.

DNA sequence has been demonstrated to be predictive of various contextual features, such as epigenetic marks and TF binding profiles, in a given cellular context [25], and hence context category of features are causally dependent on sequence category of features. In light of this, we categorized sequence features as "primary" features, while cell context features, such as epigenomic and transcriptomic features, were considered "secondary" features in our analysis. When evaluating the MPV (Mean Predictive Value) of sequence category feature families, we excluded secondary features from the models. Conversely, when assessing the MPV of cell context feature families, we included all sequence feature families in the models (see Fig. 3d). In other words, we employed "sequence-only" models to evaluate the MPV of sequence category feature families, whereas "sequence + context" models were used to calculate the MPV of cell context feature families. As mentioned earlier, there are five feature families within both the sequence and cell-context categories. Consequently, the set of sequence-only models consists of 31 models, covering all combinations of feature families in the sequence category. Similarly, there are 32 "sequence + context" models, each containing a lower dimensional representation of the full set of sequence feature families along with a combination of context feature families.

Figure 3e illustrates the calculation of MPV of feature families for prediction of Malat1-chromatin interactions. Each box represents the distribution of performance (block CV AUROC) differences between models trained using feature sets that differ only by a specific feature family. The average of the depicted distribution is the MPV of that feature family. We see that transcription is the dominant discriminative feature family in predicting Malat1-chromatin interactions: regardless of other feature families included in the models, including the transcription family increases the AUROC by ~ 0.05 on average. This is consistent with the localization of Malat1 to nuclear speckles and their known association with transcriptional activity [26, 27]. We repeated the same procedure for all 15 lncRNAs and report the MPVs as a heatmap in Fig. 3f. (Supplementary Fig. S2 shows the corresponding heatmap generated using MxPV values). We found the “Transcription” family of features to be predictive (MPV ≥ 0.01) for 9 lncRNAs, which are henceforth referred to as “Transcription-associated lncRNAs” or “TA lncRNAs”. The strongest example of this is the lncRNA Trerf1, for which the Transcription family has an MPV of 0.1. Figure 3g compares the ROC of a baseline sequence-only classifier (that includes all five sequence feature families) of Trerf1 interactions with that of a classifier that also includes the Transcription feature family, with the AUROC increasing by 0.11, from 0.66 to 0.77 as a result. Two other lncRNAs for which the Transcription family has similarly high MPVs are Platr16 and 5830418p13rik (Supplementary Fig. S3). The remaining six lncRNAs (rows labeled in navy, Fig. 3f) are henceforth referred to as “non- Transcription-associated lncRNAs” or “non-TA lncRNAs”. Among the context feature families, transcription is by far the most predictive feature (average MPV of 0.03 across all lncRNAs) while other feature families have relatively low predictive ability (average MPV < 0.01). Note that the low MPV assigned to ChIP profiles and chromatin marks might be due to their dependence on sequence features – our evaluation procedure is insensitive to the importance of cell-context features that are predictable from sequence. On the other hand, the high MPV attributed to Transcription family of features suggests that this feature family may bear information complementary to sequence features for prediction of lncRNA-chromatin interactions.

Interestingly, Fig. 3f suggests that the triplex family of features is predictive for several of the non-TA lncRNAs. A strong example of this is the lncRNA Rian, for which inclusion of this family increases the AUROC of various baseline models by 0.07 on average; the ROC of a classifier that only uses this feature family is shown in Fig. 3h (AUROC 0.65). The triplex family has significantly higher MPVs for non-TA (mean 0.04) versus TA lncRNAs (mean 0.008, Mann–Whitney test *p*-value = 0.01, Fig. 3i). These findings suggest the existence of two distinct classes of lncRNAs: those interacting with chromatin through triplex structure formation and those interacting through a transcription-coupled mechanism. Figure 3f also suggests k-mer frequency, motif scan, and to a lesser extent repeat feature families to be useful predictors overall, with average MPVs of 0.03, 0.02, and 0.01 respectively. For some lncRNAs, two or more feature families were predictive (MPV >  = 0.01), an example being Gm14820, for which the families “Motif scan”, “k-mer frequency” and “Repeat element count” in the sequence category have MPVs of 0.05, 0.06 and 0.05 respectively, and the Transcription family is also predictive with an MPV of 0.04. Figure 3j depicts the ROC of classifiers using each of the above-mentioned sequence feature families, a classifier that uses all five sequence families, as well as the effect of including the Transcription feature family alongside sequence features. It shows how prediction of interacting tiles for this lncRNA benefits from sequence features as well as cell context-specific transcription information.

### Combination of DNA methylation and other epigenomic features is informative of lncRNA-chromatin interactions

We designed the MPV score to capture the predictive value of a feature family in a way that is not overly dependent on whether another feature family is also included in the model or not. As a result, this approach may not detect the predictive value of feature families that are only informative when considered together with another family. To address this issue, we assessed the degree of complementarity among feature families for a given lncRNA, by comparing the predictive ability of a pair of feature families to that of either feature family. Specifically, we compared the average block CV AUROC of the model trained using a pair of feature families, with the maximum AUROC achieved using either of the feature families as the sole predictor. A gap in the compared AUROC values argues for the complementarity of information encoded by the pair of families. Hence, we call the calculated difference in AUROCs the “complementarity score” of the examined feature family pair. The result of this analysis for the family pairs with highest mean complementarity score, averaged over all considered lncRNAs, is reported in Fig. 3k. (Also see Supplementary Fig. S4.a.) The three highest scoring pairs were (Methylation, Chromatin marks), (Methylation, TF ChIP) and (Methylation, Accessibility), each involving Methylation and another epigenomic feature family. An example of such complementarity is shown in Fig. 3l, which depicts ROCs for models predicting Neat1-chromatin interactions using DNA methylation or chromatin marks only or using both feature families. The AUROC increases by 0.09 (to 0.73) when using both features together (see Supplementary fig. S4 b,c, for additional examples). Interestingly, DNA methylation and Chromatin marks together predict chromatin interactions of TA lncRNAs significantly better than that of non-TA ones (Wilcoxon *p*-value 3e-5, Fig. 3m). These observations suggest that DNA methylation, in combination with other chromatin states, may play an important role in lncRNA-chromatin interactions (see Discussion). Note that DNA methylation was not found to be informative from MPV analysis (Fig. 3f) and its combination with other features was crucial in recognizing its potential role.

### Fine grained analysis of predictive features suggests global mechanisms of lncRNA-chromatin interaction

We next sought to identify features predictive of lncRNA-chromatin interactions at the finest resolution, going beyond feature families such as “TF ChIP” to individual features such as a particular TF’s DNA-binding (ChIP) profile. To rank the features of each model by their importance, we used the “Gini-index”, commonly used with RF models. Since we had trained numerous RF models (classifiers) for each lncRNA, using different combinations of feature families, we now considered only the top 20 percentile of all models (by accuracy), and extracted a feature’s rank by its importance for each of these models. This yielded a distribution of importance ranks for each feature, in the context of each lncRNA. The importance ranks of sequence and context features were obtained separately from the sequence-only models and the “sequence + context” models respectively, and are not comparable across the two feature categories.

**Fig. 4:**
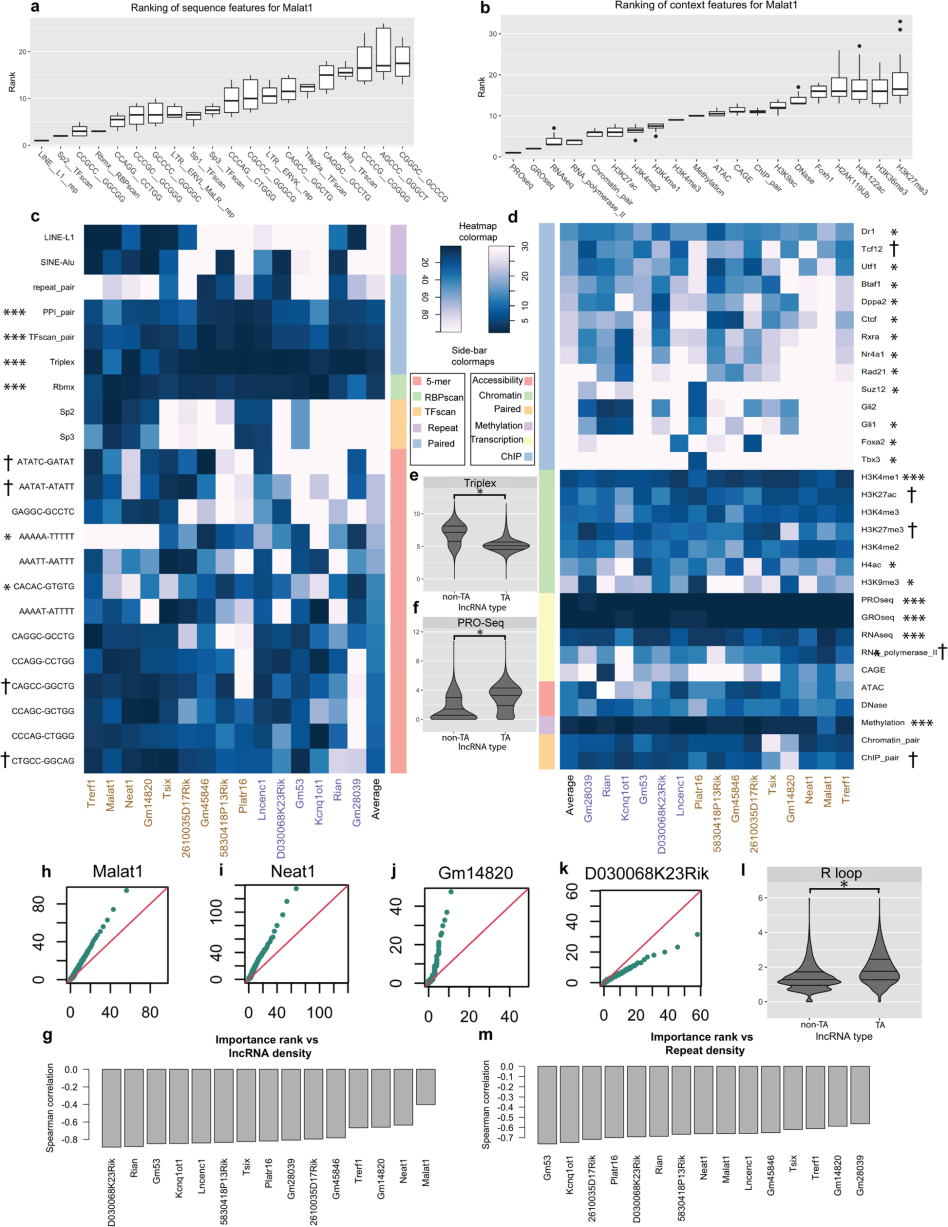
Individual feature importance analysis. **a** Importance ranks of top 20 sequence features for prediction of Malat1-chromatin interactions. Each boxplot shows the distribution of importance ranks of a feature across a set of models. The selected models are the best performing sequence-only models. **b** Importance ranks of top 20 context features according to the best performing sequence-context models trained to predict Malat1-chromatin interactions. **c** Heatmap illustrates the median importance rank of sequence features (rows) for all lncRNAs (columns) in sequence-only models that achieve top 20 percentile test performance. Side-bar colors distinguish the family of the shown features. **d** Similar to c, heatmap illustrates the median importance ranking of context features in models that achieve top 20 percentile test performance. As an example, the median values of the boxes shown in panel b are used to populate the column corresponding to Malat1. Features that are among the ten most important for all lncRNAs are marked with ***, whereas the features that are only important for at most three lncRNAs are marked with *. Additionally, features with significantly different rankings (Wilcoxon rank sum test *p*-value < 0.05) between TA and non-TA lncRNAs are marked with †. Panels **e**, **f** illustrate the distribution of Triplex (**e**) and PRO-Seq (**f**) feature values in tiles bound by lncRNAs, grouped by the lncRNA type. In both cases, a *p*-value of less than 2.2e-16 is obtained by Kolmogorov–Smirnov test. **g** Correlation analysis between number of occurrences of 5-mers in a lncRNA’s sequence and the importance rank of the 5-mer as obtained by the Random Forest classifier. Panels **h**, **i**, **j**, and **k** illustrate q-q plots comparing the distribution of R-loops in tiles bound (y-axis) versus unbound (x-axis) by Malat1, Neat1, Gm14820, and D030068K23Rik, respectively. **l** Comparison of R-loop distribution in tiles bound by TA lncRNAs versus the ones bound by non-TA lncRNAs. A *p*-value of less than 2.2 e-16 is obtained by Kolmogorov–Smirnov test. **m** Correlation analysis between average density of k-mers in consensus repeat element sequences obtained from Dfam and the importance rank of the 5-mers as obtained by the Random Forest classifier

Figure 4a,b show the 20 sequence and cell-context features respectively with highest median rank for the prediction of Malat1-DNA interactions. Prominent among the predictive sequence features are putative binding sites (motif scans) for SP family of TFs (Sp1, Sp2, Sp3) and the RBP Rbmx (also known as HnRNPG), as well as CG-rich k-mers, suggesting mechanisms related to transcription [28, 29]. This is consistent with the literature reports that Malat1 is localized to nuclear speckles [26] and interacts with nascent RNA through protein complexes [30]. The highest-ranking context features include PRO-Seq, GRO-Seq and RNA-Seq measurements at the tiles, further supporting a role for transcription in chromatin interactions of this lncRNA (see Discussion for comments on other high-ranking features).

Figure 4c,d illustrate the median importance rank of a selected group of features from sequence and cell-context categories respectively, for each lncRNA. Among sequence features informative (top 10 of 1,280, by importance) for all lncRNAs, we find features that couple the sequence information of lncRNA and DNA tiles: “PPI-pair” (number of protein complexes with predicted sequence affinity for both DNA tile and lncRNA) and “TFscan-pair” (number of TFs with predicted sequence affinity for DNA tile and lncRNA gene). This suggests a shared affinity of lncRNA and the DNA tile for individual proteins or protein-complexes as a potential mechanism mediating their interaction [31]. Another interaction mechanism suggested by this analysis is triplex formation, which is among the sequence features informative for all lncRNAs. Triplex formation has been noted previously as a likely chromatin-interaction mechanism for a variety of lncRNAs [6, 32]. The only other sequence feature that is important for every considered lncRNA is the predicted affinity of the theoretical transcript corresponding to the DNA tile for the RBP Rbmx. Rbmx is known to function in multiple cellular processes including RNA splicing, miRNA processing, methylation and DNA Damage Response (DDR) [33–37]. It is known as a reader of m6A, an abundant RNA modification [38]. Malat1 harbors m6A modification and interacts with Rbmx in a way that is dependent on the status of this RNA modification [29]. Rbmx has been shown to interact with the lncRNA NORAD and that interaction is important for the role of NORAD in maintaining genome stability [39]. Among the consistently informative cell-context features (among top 10 of 333, for each lncRNA), marked with *** in Fig. 4d, we find features associated with transcriptionally active states including PRO-Seq, GRO-Seq, RNA-Seq, and H3K4me1, supporting a strong connection between lncRNA-chromatin interaction and transcriptional activity.

Considering the observed importance of triplex formation potential (as captured by triplex sequence feature) and nascent transcription (as captured by PRO-Seq context feature) for all lncRNAs, we investigated their distribution in DNA tiles bound by TA lncRNAs as compared to non-TA lncRNAs. Figure 4e reveals that DNA tiles bound by non-TA lncRNAs exhibit significantly higher triplex formation potential with their corresponding lncRNAs (Kolmogorov–Smirnov test, *p*-value < 2.2 e-16). Conversely, tiles bound by TA lncRNAs give rise to significantly higher number of nascent transcripts as compared to the tiles bound by non-TA lncRNAs (Fig. 4f, *p*-value < 2.2 e-16). These observations reaffirm similar findings reported above using feature family-level analysis and complementary statistical methods. (Comparisons for all other sequence and context features are reported in Supplementary Figs. S5 and S6, respectively.)

Going beyond features informative for all or most lncRNAs, we noted several C/G-rich 5-mers are predictive (among top 10 by importance rank) for two or more lncRNAs (Fig. 4c). The six 5-mers with highest average importance across considered lncRNAs are CTGCC, CTGGG, GCTGG, GGCTG, CCTGG and GCCTG, each composed of four C/G nucleotides and one A/T nucleotide while lacking the CG dinucleotide. Interestingly, such a k-mer composition has been observed in RNA sequences involved in RNA nuclear localization [40–42], but in our analysis the pattern was found to distinguish bound and unbound DNA tiles. Inspired by this finding, we explored the relationship between lncRNA and DNA tile sequences. As shown in Fig. 4g, for all of the considered lncRNAs, we found a significant negative correlation (Spearman *p*-value < 2.2e-16) between the frequency of 5-mers in lncRNA sequence and their importance ranking in prediction of chromatin interactions of that lncRNA. Identification of the same patterns on nucleus-enriched lncRNAs and their bound DNA tiles is intriguing: a possible explanation is that nuclear localization of these lncRNAs is mediated by proteins that bind to the specific sequence pattern in the lncRNA as well as nascent transcripts on chromatin [43]. This explanation is supported by the consistently high importance ranking obtained for ‘PPI pair’ and ‘TFscan pair’ features that belong to ‘Shared motif’ family (Fig. 4c). Another possible explanation for this finding is the formation of R-loops between lncRNAs and template DNA through sequence complementarity [44]. To test this explanation, we compared the distribution of R-loops between bound and unbound tiles using publicly available DRIP-Seq data on mESC from [45]. Figure 4h-k illustrate a pronounced difference in the distribution of R-loops in bound vs unbound tiles for Malat1, Neat1, GM14820, and D030068K23Rik. This statistical trend was observed consistently, with significant (Kolmogorov–Smirnov test, adjusted *p*-value < 2.2e-4) differences between bound and unbound tiles for 15 out of 16 considered lncRNAs. We also noted that consistent with the reported association R-loops with transcription [46], tiles bound by TA lncRNAs have significantly higher levels of R-loop formation as compared to non-TA ones (Fig. 4l).

We observed above in Fig. 4c that the occurrence of repeat elements Alu and L1 is informative about chromatin interactions of a subset of lncRNAs, including Neat1, Malat1, and Rian. Alu element has been previously linked to nuclear retention of lncRNAs [41]. This finding, together with the predictive value of other repeat elements for lncRNAs, such as the importance of MalR and ERVK long terminal repeats (LTRs) in predicting Malat1 interactions, led us to further investigate the relationship between repeat elements and lncRNA-chromatin interactions. Figure 4m shows the correlation between average density of 5-mers in all Dfam consensus repeat elements [47] and their importance ranking in prediction of chromatin interactions; the correlation values are statistically significant (*p*-value < 2.2e-16) for every lncRNA examined.

The importance of a feature as determined by the RF models does not inform us about the enrichment versus depletion of that feature in lncRNA-bound DNA tiles. To address this, we directly inspected their distribution in bound versus unbound tiles (see Supplementary Fig. S7 and Supplementary Note 1). We noted, as expected, that nascent transcription (represented by PRO-Seq feature) is enriched in the DNA tiles bound by TA lncRNAs Malat1, Neat1, Gm14820, and Trerf1, and depleted in the tiles of non-TA lncRNAs Gm53 and D0300068k23Rik. Interestingly, the Alu and Line-1 repeats, both found to be important features in Fig. 4c, show trends opposite to each other, with enrichments in regions bound by TA and non-TA lncRNAs respectively. Line-1 repeats present an intriguing case: they emerged as the most important sequence feature for Malat-1 (Fig. 4a), a TA lncRNA, but are otherwise enriched in interactions of non-TA lncRNAs.

In summary, our fine-grained feature importance analysis suggests global mechanisms, including transcription, triplex/R-loop formation, and protein binding, likely to be involved in chromatin interactions of many lncRNAs.

### Feature analysis links lncRNA-chromatin interactions to DNA methylation and DNA Damage Response

We noted above (Fig. 4c) that a set of 5-mers are predictive of chromatin interactions of multiple lncRNAs. These 5-mers include the CAG/CTG trinucleotide while lacking the CG dinucleotide. CAG trinucleotides are prone to non-CpG methylation and are observed to be particularly methylated in neurons and ESCs [48, 49]. This raises the possibility of DNA methylation being involved in lncRNA-chromatin interactions, a hypothesis further supported by the observation that experimentally measured DNA methylation is important to classifiers for all lncRNAs (Fig. 4d).

The above-mentioned 5-mers (GCTGG, CCTGG, etc.) are not only methylation related, they are also hotspots of DNA recombination [50–52] and associated with DNA Damage Response (DDR). Cytosine methylation and DDR are closely related processes and DNA methyltransferases have been implicated in DDR [53]. A closer examination of the important features (Fig. 4c,d) revealed several additional features related to DDR to be differentially present in bound versus unbound tiles of multiple lncRNAs (Supplementary fig. S8, Supplementary Note 2). These include the histone mark H2A.Z, known to be required for double strand break repair [54], histone modifications H3K27me3 and H2AK119Ub that are associated with DDR [55, 56], DNA binding affinity for Pparγ, a nuclear receptor TF involved in DDR [57], the RBP Rbmx that was recently found to be essential to DDR [33], among others. A DDR-related TF named Mecp2 was found to have its predicted binding sites (motif scan feature) enriched in positive versus negative tiles of Malat1 (*p*-value < 2.2e-16) and Neat1 (*p*-value 1e-4). Mecp2 has been reported to interact with Neat1 [58] and Malat1 [59] among other lncRNAs, and is known to bind methylated cytosine, specifically in non-CpG context [49]. Other proteins involved in methylation and/or DDR, including Asxl1 [60, 61], Pcgf1 [62], Rad21 [63] and Tdg [53] were noted as having enrichment of binding (TF ChIP feature) in positive versus negative DNA tiles of Malat1, Neat1 and other lncRNAs.

In other words, our analyses identified several features, from both the sequence and cell-context categories, implicating methylation and components of the DNA damage repair program in lncRNA-chromatin interactions.

### MicroRNA seed sequences distinguish lncRNA-associated DNA segments

**Fig. 5:**
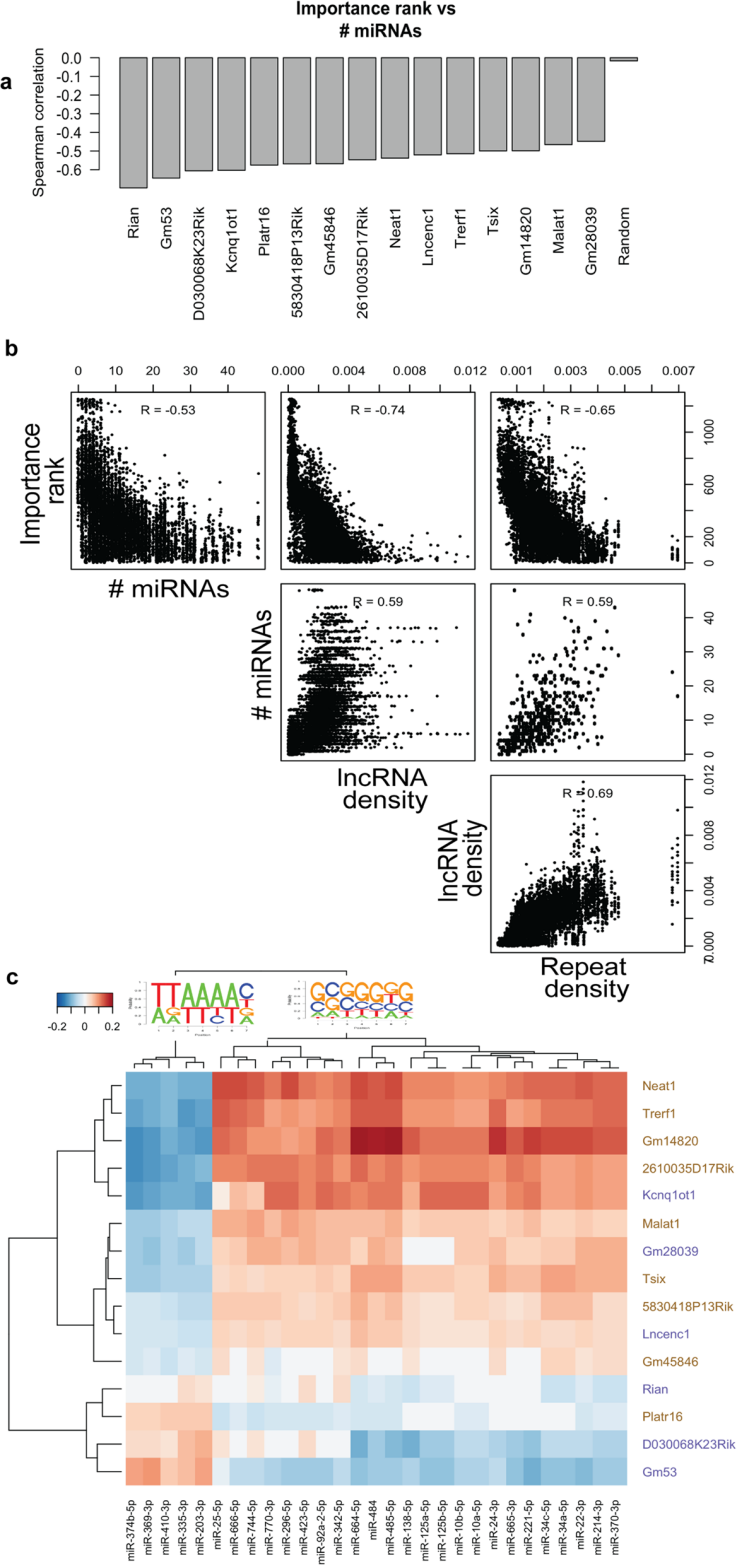
Potential role of miRNAs in lncRNA-chromatin interactions. **a** Correlation analysis between number of miRNAs whose seed sequence contains the 5-mer and the importance rank of the 5-mer as obtained by the Random Forest classifier for a specific lncRNA. **b** Each scatter plot corresponds to the pair of entities indicated in left-most and lower-most panels. In all panels except rightmost panel in the middle row, each point represents a 5-mer-lncRNA combination. In rightmost panel in the middle row each point represents a 5-mer. Spearman correlation is shown at the top of each panel. **c** Signed Kolmogorov–Smirnov statistic heatmap for a selection of miRNAs known to be expressed in mESC. Each row represents a lncRNA and each column represents a miRNA. Sequence logo indicating the seed sequence composition of each of the two top-level clusters of miRNAs is drawn on the dendrogram

Further investigation of the 5-mers identified as important for prediction of lncRNA-chromatin interactions revealed that several of these 5-mers appear in seed sequences of mouse miRNAs. This observation intrigued us, as lncRNAs are known to function, in some cases, by binding to miRNAs and sequestering them away from their regulatory targets (“competing endogenous RNA” [16]). Similar to the above-mentioned correlations between importance rank of k-mers and their density in lncRNAs (Fig. 4g) and repeat elements (Fig. 4m), we noted a high correlation between the number of times a 5-mer appears in miRNA seed sequences and its importance rank in our lncRNA-chromatin interaction prediction models (Fig. 5a). To further investigate this phenomenon, we directly quantified the relative enrichment of the 5-mers comprising miRNA seed sequences in bound versus unbound DNA tiles of each lncRNA, by computing a measure called “signed K-S statistic” (see Methods). In Fig. 5c, we illustrate this measure for a selection of miRNAs known to be expressed in mESC [64]. Hierarchical clustering of the heatmap depicts two main groups of miRNAs, with G/C-rich and A/T-rich seed sequences respectively (motifs shown in figure). These two groups of miRNA seed sequences are statistically associated with two distinct groups of lncRNAs in terms of their utility for predicting lncRNA-chromatin interactions.

Figure 5b illustrates the relationship between importance rank of k-mers to lncRNA-chromatin interactions and their density in lncRNAs, miRNA seed sequences, and repeat elements. The significant correlation between all pairs of the considered entities is intriguing and may point to a network of nucleic acid interactions centered around specific k-mers that are enriched in non-coding RNAs and repeat elements.

Taken together, the results above suggest that signatures of miRNA-mRNA interaction (miRNA seed sequences) are also associated with lncRNA-chromatin interactions. Such an association is not necessarily expected to result from the widely studied ceRNA mechanism, since lncRNA-miRNA interactions in this mechanism are not localized at the chromatin. We believe it to be evidence of mechanisms involving physical interactions among miRNAs, lncRNAs and DNA, that merit future investigation.

## Discussion

In light of the RNA-world hypothesis [65], it is conceivable for RNAs to play major roles in the most primary cellular functions including organization, transcription, and maintenance of DNA. Such fundamental roles have been identified for several RNA species. For example, X chromosome inactivation was found to be carried out in part by a lncRNA, named XIST [66]. Many other lncRNAs are found to be enriched in the nucleus and interact with the chromatin. Interactions between RNA and chromatin have recently been recognized to serve a variety of functions including regulation of transcription and splicing, as well as chromatin organization [31, 67–69]. However, neither the underlying mechanism nor the precise function of specific RNA-chromatin interactions is resolved. In this work, we examined genome-wide RNA-chromatin interaction data through machine learning techniques, to identify distinctive patterns characterizing the RNA-interacting genomic regions.

The first methodological challenge we faced was that interactions are predominantly limited to regions near the lncRNA gene and a standard classification framework would mainly rely on this signal (and other locally frequent features) to achieve high discriminative power. We used a carefully defined negative set and a specialized cross-validation scheme to force the models to reveal additional signals. We then considered a multitude of features describing genomic regions and attempted to carve out the most informative of these features at different levels of granularity. We grouped individual features into feature families reflecting the nature of their information (e.g., TF ChIP-seq or repeat element counts), and further categorized the feature families depending on whether they provide information on sequence or the cellular context. To quantify the relevant information content of each feature family, we trained Random Forest models to predict RNA-chromatin interactions using each feature family in presence or absence of every other one. In this way, the predictive value of feature families is quantified while accounting for their interdependence. This approach is inspired by recent advances in techniques for quantifying feature importance in machine learning models [23, 24], and was necessitated in our analyses by the extensive correlations among different epigenomic features. Our categorization of features into “primary” (sequence-based) and “secondary” (contextual) was motivated by the fact that sequence at least partially determines epigenomic states, and predictive contextual features may thus be redundant with their underlying sequence footprints. Finally, the best performing models were dissected using standard machine learning techniques to identify the most informative individual features.

We found the feature families of transcription, k-mer frequencies and triplex formation potential to have the most predictive value overall (Fig. 3f), whereas DNA methylation was found to contain predictive information complementary to that of chromatin marks, TF binding and DNA accessibility (Fig. 3k). We also note that while DNA methylation emerged as an important feature in the assessment of individual features, it was not revealed as important in the MPV-based analysis. This is due to the conceptual differences in the two analysis: the MPV-based analysis may underestimate the significance of a feature (or feature family) if it is informative only when considered together with another feature. This limitation of the MPV approach, notwithstanding its other strengths, led us to explore and discover the complementarity between methylation and other features such as chromatin marks, TF ChIP and accessibility. At the same time, the methylation feature, defined here as a simple sum of the methylation scores of all cytosines in a segment, is admittedly a simplistic one, given that the impact of methylation can be context-dependent. Future studies may explore use of richer representations of local DNA methylation in assessing the latter’s role in lncRNA-chromatin interactions.

K-mer frequencies were predictive of interactions for all considered lncRNAs, consistent with reports that functionally related lncRNAs have similar k-mer profiles [70]. In contrast, transcription and triplex formation potential were found to predictive only for distinct groups of lncRNAs, which we termed transcription-associated (TA) and non-TA groups respectively. This finding suggests distinct mechanisms of action for the two groups: non-TA lncRNAs putatively bind to DNA and inhibit transcription by forming stable triplex structures, whereas TA lncRNAs are involved in transcription in a sequence-dependent way. The interaction between non-TA lncRNAs and chromatin may be partly mediated by specific protein complexes, as evidenced by a significantly higher importance rank for the “ChIP-Pair” feature (Fig. 4d) as well as high importance ranks for protein complexes such as Gli1/2 for this group of lncRNAs. On the other hand, interactions of TA lncRNAs may in part be mediated through formation of R-loops between GC rich sequences in transcriptionally active genomic regions and their complementary sequences on the lncRNA. This is supported by significant enrichment of experimentally profiled R-loops (DRIP-seq) in lncRNA-bound regions, especially for TA lncRNAs (Fig. 4h-l).

Investigating sequence features at a higher resolution (i.e., individual feature analysis), we found shared motifs (PPI-pair, TFscan-pair) to be an important feature for all the considered lncRNAs (Fig. 4c). This is consistent with the enrichment of classifier-identified informative k-mers in lncRNA sequences (Fig. 4g), and both point to a sequence-based interaction mechanism. This mechanism could be directly mediated through formation of R-loops (mainly for TA lncRNAs) or be indirectly mediated by protein complexes present in the microenvironment (both TA and non-TA lncRNAs).

Formation of trans R-loops between lncRNA and transcribed DNA has been previously reported [7, 44]. This phenomenon may be a mechanism for the known localization of certain lncRNAs, notably Malat1 and Neat1, to membrane-less organelles such as nuclear speckles and paraspeckles [71]. For instance, the functional link between MALAT1 and nuclear speckles — alternative splicing — is dependent on G quadruplex (GQ) structures in the 3’ region of MALAT1 [72], and genomic regions enriched with GQs are known to preferentially give rise to R-loops [73]. Also, there is emerging evidence that several proteins that interact with R-loops, and contain Intrinsically Disordered Regions (IDR), undergo liquid–liquid phase separation characteristic of membrane-less organelles in the cell nucleus [74]. Interestingly, we also found putative examples of lncRNA-chromatin interactions mediated by RBPs, many of which, including Rbmx (one of our most informative features, Fig. 4c) harbor IDRs [75]. In light of the above observations, we speculate that membrane-less organelles are at least in part shaped through trans R-loop formation between transcribing DNA and lncRNAs, and further stabilized through multi-valent interactions mediated by IDR-containing RBPs recruited by the lncRNAs. Moreover, the model-predicted importance of features such as nascent transcription (e.g. GRO-Seq, PRO-Seq), count of recombinogenic 5-mers (e.g. GCTGG, CCAGG [76]), and markers of DNA repair (e.g., H2AZ [54], H2AK119Ub [77] and components of NuRD complex [78]) suggests that lncRNA-chromatin interactions may carry out functions (or form the scaffold of a microenvironment) related to transcription, recombination, and DNA repair, all of which are associated with R-loops [79, 80].

We found that 5-mers prone to Double Stranded Break (DSB) – C[C/G][A/T]GG – are enriched in lncRNA sequences, lncRNA-interacting DNA segments, as well as miRNA seed sequences. This observation does not have an obvious explanation, as the well-known lncRNA-miRNA relationships (an aspect of competing endogenous RNAs) do not demand sequence footprints in the DNA. Interestingly, a DNA repair mechanism has been identified involving miRNA-sized DNA Damage RNA (DDRNA) and lncRNA-sized damage-induced lncRNAs (dilncRNAs), and interactions between DDRNA and dilncRNAs seem to be essential in the repair process [81–83]. Taken together, perhaps these findings and our statistical observations point to a role for specialized miRNAs and lncRNAs in counteracting the frequent DSB at genomic locations harboring these particular k-mers.

The blocked structure of enrichment of miRNA seed sequence 5-mers in tiles bound by lncRNAs (Fig. 5c) was a surprising observation. It reveals distinct groups of miRNAs and corresponding groups of lncRNAs such that the miRNA seed sequences in each group are enriched in the DNA tiles bound to lncRNAs in the matching group but depleted in (tiles of) the other group of lncRNAs. This may hint at competing modules of interacting entities (miRNAs, lncRNAs, DNA segments), each regulating the formation of microenvironments suited for specific functions such as transcriptional response to environmental stimuli. Speculating further, one possibility is that particular miRNAs have evolved to counteract the R-loop formation by binding to lncRNAs, in a way similar to the sponging phenomenon observed in ceRNA mechanism, and de-activating the lncRNA, hence, preventing the formation of R-loops. In this way lncRNAs and miRNAs may together regulate transcription and DNA repair by regulating the formation of R-loop in particular genomic regions (e.g., repetitive elements) that are enriched in the same k-mers.

We identified nascent transcription as one of the most important features in predicting lncRNA-chromatin interactions. One possible role for transcription-associated interactions is the formation of context-specific transcription factories [84]. LncRNAs together with IDR-containing RBPs (e.g., Rbmx) may act as scaffolds bringing together a specific set of TFs and the transcription machinery.

It is interesting that methylation is relatively higher in tiles bound by TA lncRNAs as compared to non-TA ones (Fig. S5), since DNA methylation is generally associated with decreased expression. However, non-CpG methylation is observed to be associated with increased expression in neuronal cells [49]. It is interesting to note that CC[A/T]G[G/C] is known as the best substrate for non-CpG methylation in ESC and neuronal cells [51]. (This is almost identical to the DSB-prone 5-mer noted above as being enriched in lncRNAs, their interacting tiles as well as miRNA seed sequences.) Hence the higher methylation rate in tiles bound by TA-associated lncRNAs maybe due to non-CpG methylation. Indeed, lncRNA and miRNAs are disturbed in diseases related to methylated cytosine [59], particularly in Rett’s syndrome and other diseases related to Mecp2 which is known as the exclusive reader of non-CpG methylation [85]. Another possible explanation of the greater methylation levels in tiles bound by TA lncRNAs is that these tiles include gene bodies, which are known to be associated with high methylation levels [86].

One caveat of our analysis is that GRID-Seq and RADICL-Seq experiments capture protein mediated interactions through crosslinking and hence direct RNA–DNA interactions (i.e., those not mediated by proteins) are not captured by these methods [13]. This limits the mechanistic and functional insights accessible by our analyses, although we were still able to recover a strong role for triplex formation and R-loop formation. It is also noteworthy that at least one of the analyzed lncRNAs – Neat1 – is not yet fully active at this embryonic stage and its wide range of trans interactions often observed in other cell-types are absent in the data analyzed by us.

We note that our analysis focused on determinants of lncRNA-chromatin interaction from the perspective of the genome, i.e., why certain segments are lncRNA-bound while others are not. Since each classifier was trained for a single lncRNA, we did not stand to gain from utilizing information on the lncRNA’s expression level. Such information can be useful in understanding lncRNA mechanisms and functions, as is evidenced from prior studies on lncRNA co-expression networks [87]. For instance, in light of the observed potential role for miRNAs in lncRNA-chromatin interactions, investigating the correlations between expression levels of lncRNA and miRNAs with similar sequence features (e.g., 5-mer frequencies) may shed light on the nature of their potential interactions.

We relied on Random Forests as classifiers in this study because of their general reputation for good performance in a variety of contexts and relative ease of interpretation of trained models. Exploration of alternative classification models in the future may result in better fits and potentially new insights into the factors underlying lncRNA-chromatin interactions.

We finally note that the cell-context features found to be important in this study only apply to embryonic stem cells. A more comprehensive study of lncRNA-chromatin interaction across multiple cell types might reveal more insights into the context determinants of lncRNA-chromatin interactions.

## Methods

### Features used in classification

We aimed to perform an unbiased investigation to uncover characteristics that distinguish DNA tiles that interact with a particular lncRNA. The following families of features were used to describe each DNA tile.

*Sequence category:*

- k-mer frequency: Consists of 512 features describing the DNA tile, each specifying the number of times a 5-mer or its reverse complement occur in the 1-kb DNA tile. We used Jellyfish [88] to obtain the count of all 1024 possible 5-mers on every tile.
- Motif scan: We used PWMScan [89] to scan the mouse genome, tiled into 1-kb long segments, for hits of directly identified (as opposed to inferred) mouse TF (623 unique TFs from [90] were considered) and RBP (78 unique RBPs from [91] were considered) PWMs from Cis-bp database. Motif hits with *p*-value larger than 1e-4 were filtered out. For each DNA binding protein, overlapping motif hits from all its different motifs were merged using bedtools “merge” command with distance threshold set to 1 bp [92]. The number of motif hits after merge for each DNA/RNA-binding protein was used as its feature value. Note that the tile’s putative transcript was scanned for RBP motifs.
- Triplex formation potential: Triplexator [93] was used to compute the triplex formation potential between a given lncRNA and any DNA tile. Parameters were chosen based on recommended settings in [94]. We used the summary output (–output-format 2), comprised of four values describing the relative triplex formation potential between each lncRNA and DNA tile, as features in our models.
- Shared motifs: This family comprises three features. “TF pair” indicates the number of unique TFs with at least one motif hit on both the lncRNA gene and the DNA tile. Similarly, “RBP pair” is the number of unique RBPs with at least one motif hit on the putative transcript of the DNA tile as well as the lncRNA. Finally, “PPI pair” is based on mESC specific protein–protein interaction network obtained from Escape database [95]. This feature represents the number of unique interacting protein-pairs such that one of the interacting proteins is predicted to bind the lncRNA and the other is predicted to interact with the DNA tile or its putative transcript). For example, if a lncRNA is predicted to bind a particular RBP that interacts with a TF with putative binding sites on a DNA tile, the RBP-TF pair is counted in calculating the ‘PPI pair’ feature. The motifs used in this analysis and the motif scanning method are described in “Motif scan” section above. Shared motifs feature family is meant to capture the number of potential mediators for the interaction between a given pair of lncRNA and DNA tile.

*Cell context category:*

- DNA accessibility: Consists of DNase Hypersensitivity profile and ATAC-seq profile of mESC obtained from ChIP-Atlas [96] and GSE113592, respectively. Maximum score of peaks overlapping the DNA tile was used as its score.
- DNA methylation: Processed data (in bedgraph format) from whole genome bisulfite sequencing experiment was obtained from [97]. Sum of the methylation score of all cytosines within a tile was used as the methylation score of the tile.
- Chromatin marks: ChIP-Seq data from 48 unique histone marks profiled in mESCs was obtained from ChIP-Atlas [96]. Downloaded from: http://dbarchive.biosciencedbc.jp/kyushu-u/mm9/allPeaks_light/allPeaks_light.mm9.05.bed.gz on Aug 27, 2020. ChIP peak scores from multiple experiments with the same antigen were combined by identifying peaks overlapping with each DNA tile and assigning the maximum of their scores to the tile.
- TF ChIP: ChIP-Seq profiles of 275 proteins in mESC obtained from ChIP-Atlas dataset. Processed ChIP-Seq data for mESC cell line was downloaded from ChIP-Atlas database: http://dbarchive.biosciencedbc.jp/kyushu-u/mm9/allPeaks_light/allPeaks_light.mm9.05.bed.gz. ChIP signals from multiple experiments with the same antigen were combined as described above.
- Transcription: Transcriptomic profile of mESC obtained from GRO-Seq [98], PRO-Seq [99], RNA-Seq [100], CAGE [101], and RNA pol II ChIP-Seq [96] experiments. GRO-Seq and PRO-Seq data were obtained as processed bigwig files. Sum of the score of nucleotides forming the1-kb DNA tile was used as its score. RNA-Seq data was downloaded as bigwig files from https://www.ncbi.nlm.nih.gov/geo/query/acc.cgi?acc=GSE29184 on Aug 12, 2020. Maximum score of windows overlapping a DNA tile was used to determine feature value. Processed CAGE data was obtained from Supplementary Dataset 2 reported in [101]. Maximum score of CAGE peaks overlapping a DNA tile was used as the CAGE feature score for the tile. RNA pol II ChIP-Seq data was processed the same as TF ChIP features.
- Paired context features: ChIP-pair and Chromatin-pair features belong to TF ChIP, Chromatin marks family of features, respectively. ChIP-pair quantifies the number of potentially interaction-mediating proteins: counts unique proteins with ChIP peak detected both on the lncRNA gene and the DNA tile. Chromatin-pair captures chromatin context similarity between the DNA tile and lncRNA gene: counts the number of unique chromatin marks with ChIP profiles overlapping both the DNA tile and the lncRNA gene.

### Choice of negative examples

To form the negative set of datapoints for the classification task, each negative tile (i.e., tiles that do not interact with the lncRNA) in the genome was assigned to its closest positive tile. Next, negative tiles were filtered such that the number of negative tiles with the same assigned unique closest positive tile is limited to one hundred. This constraint limits the concentration of negative tiles in vicinity of any particular positive tile. Next, the distribution of distances for all positive tiles to the lncRNA gene was computed. This distribution was used to construct the sampling probability vector for the negative tiles based on their distance to the lncRNA gene. The number of negative tiles was limited to five times the number of positive tiles (except for Malat1, with negative tiles being twice the number of positive tiles). The number of positive and negative tiles chosen for each lncRNA is reported in Supplementary Table 1.

### Block cross-validation

Random and block CV strategies were applied for dividing tiles into five non-overlapping partitions. In block partitioning mode, first all negative points were assigned to their closest positive example. Next, a number of seed positive examples were randomly selected to form the initial points of each partition. We put together each seed positive tile with a number of its neighboring positive tiles. The set of all seed tiles and their neighboring positive tiles form the positive examples of this partition. All the negative examples assigned to the selected positive examples complement the partition with negative tiles.

### Visualization

ggplot2, ggplots, and Gviz [102] packages in the R statistical software were used for visualizations.

### Random forest

R package ranger [103] was used for Random Forest classification. Probability trees with mean leaf node size of 10 were used. Maximum tree depth was set to 10 and square root of the number of features was used as the “mtry” parameter. Each forest consisted of 1000 trees. Up-sampling was performed to deal with class imbalance. Note that in training Sequence-Context models, sequence features are represented using their first 100 principal components (PCs). PCs are computed using R irbla package.

### Marginal predictive value computation for feature families

Considering feature families ${A}_{1}, \dots ,{A}_{n}$, we train ${2}^{n} -1$ Random Forest models with all possible combinations of feature families where at least one feature family is included. The set of ${2}^{n-1}$ models whose training features included family ${A}_{i}$ is denoted as ${E}_{i}$. $w\left(U\right)$ indicates a Random Forest model that is trained with features included in set U, and ${M}_{k}(U)$ denotes the test performance (block AUROC) of $w\left(U\right)$ on cross validation fold $k$.

$${E}_{i}=\{w\left({A}_{i }\cup B\right)| B\in P({A}_{1}, \dots ,{A}_{i-1},{A}_{i+1}, \dots ,{A}_{n})\}$$

where $P\left(S\right)$ is the power set of set $S$. We calculate Marginal Predictive Value (MPV) ${V}_{i}$ of feature family ${A}_{i}$ as follows:

$${V}_{i}= \frac{1}{{2}^{n-1}} \sum_{U \in {E}_{i}}\frac{1}{K}\sum_{k=1}^{K}{M}_{k}\left(U\right)-{M}_{k}(U-{A}_{i})$$

where $K$ is the number of cross-validation partitions.

However, in the presence of redundant features, ${V}_{i}$ of all feature families in the redundant set decreases with increasing size of the redundant set. To gain a complementary view of the performance improvement caused by ${A}_{i}$, we also compute MxPV, denoted here as ${V\mathrm{^{\prime}}}_{i}$ and computed as follows.

$${V\mathrm{^{\prime}}}_{i}= \underset{U \in {E}_{i}}{\mathrm{max}}\frac{1}{K}\sum_{k=1}^{K}{M}_{k}\left(U\right)-{M}_{k}(U-{A}_{i})$$

Figure 3d illustrates an example scenario with two primary (A and B) and two secondary (C and D) families of features. To evaluate the MPV of a primary feature family, models are trained with all possible combinations of primary features (i.e., M_A_, M_B_, M_AB_), and the MPV is calculated by averaging over the differences in test performance of models that differ only on that particular primary feature. (For instance, M_ABC_—M_AB_ is the difference in test performance of a model trained with A,B,C as features and a model trained with A,B as features.) MPV evaluation of a secondary feature family follows a similar but slightly different process, the difference being that all the primary features are included in all the models trained for the purpose of secondary feature MPV evaluation.

### Signed K-S statistic

Two-sample Kolmogorov–Smirnov (K-S) Test was performed with a two-sided alternative hypothesis. K-S test statistic is computed as the maximum absolute difference in the cumulative curve of the two samples. However, in order to gain information on the direction of difference between the two samples, we computed Signed K-S statistic as the maximum absolute difference in the cumulative curves multiplied by the sign of the difference.

## Supplementary Information

**Additional file 1: Figure S1.** Example of model predictions for Gm14820 together with predictive feature values. **Figure S2.** Maximal Predictive Value (MxPV). Heatmap shows MxPV of feature familes (columns) for all lncRNAs (rows). **Figure S3.** Transcription feature family enhances model predictions. **Figure S4.** Feature family complementarity. **Figure S5.** Violin plots showing distributions of selected sequence features in tiles bound by Transcription-associated (TA) lncRNAs and non-TA lncRNAs. **Figure S6.** Violin plots showing distributions of selected context features in tiles bound by TA lncRNAs and non-TA lncRNAs. **Figure S7.** Visualization of features distinguishing bound vs non-bound tiles for selected lncRNAs. **Figure S8 (previous page).** Visualization of DNA Damage Response- and methylation- related features. **Supplementary Table 1.** Number of datapoints each representing a 1 kb DNA tile is listed in the positive (bound) and negative (unbound) sets for each of the studied lncRNAs. **Supplementary Table 2.** **Supplementary note 1.** **Supplementary note 2.** A closer look at methylation and DDR-related factors.

## Data Availability

The datasets analyzed during the current study are reported in Methods section. Accession numbers and sources are reported in Supplementary table 2. The processed dataset used to train the Random Forest models is available at [104].
