# Supplementary material for: Integrative modeling of lncRNA-chromatin interaction maps reveals diverse mechanisms of nuclear retention
Source: BMC Genomics. 2023 Jul 13;24:395. doi: 10.1186/s12864-023-09498-9 (PMC10347723; doi:10.1186/s12864-023-09498-9)
Supplement: Supplementary file 1 — Additional file 1: Figure S1. Example of model predictions for Gm14820 together with predictive feature values. Figure S2. Maximal Predictive Value (MxPV). Heatmap shows MxPV of feature familes (columns) for all lncRNAs (rows). Figure S3. Transcription feature family enhances model predictions. Figure S4. Feature family complementarity. Figure S5. Violin plots showing distributions of selected sequence features in tiles bound by Transcription-associated (TA) lncRNAs and non-TA lncRNAs. Figure S6. Violin plots showing distributions of selected context features in tiles bound by TA lncRNAs and non-TA lncRNAs. Figure S7. Visualization of features distinguishing bound vs non-bound tiles for selected lncRNAs. Figure S8 (previous page). Visualization of DNA Damage Response- and methylation- related features. Supplementary Table 1. Number of datapoints each representing a 1 kb DNA tile is listed in the positive (bound) and negative (unbound) sets for each of the studied lncRNAs. Supplementary Table 2. Supplementary note 1. Supplementary note 2. A closer look at methylation and DDR-related factors. [file 12864_2023_9498_MOESM1_ESM.docx]

Supplementary material for

**Integrative modeling of lncRNA-chromatin interaction maps reveals diverse mechanisms of nuclear retention**

Shayan Tabe-Bordbar, Saurabh Sinha

Shayan Tabe-Bordbar

Department of Computer Science, University of Illinois at Urbana-Champaign, Urbana, IL, United States of America. Email: tabebor2@illinois.edu

Saurabh Sinha

Department of Computer Science, Carl R. Woese Institute for Genomic Biology, Cancer Center of Illinois, University of Illinois at Urbana-Champaign, Urbana, IL, United States of America. Email: sinhas@illinois.edu

Corresponding Author:

Saurabh Sinha
2122 Siebel Center, 201 N. Goodwin Ave, Urbana, IL 61801. USA.

Phone: 217-333-3233

Email: [sinhas@illinois.edu](mailto:sinhas@illinois.edu)


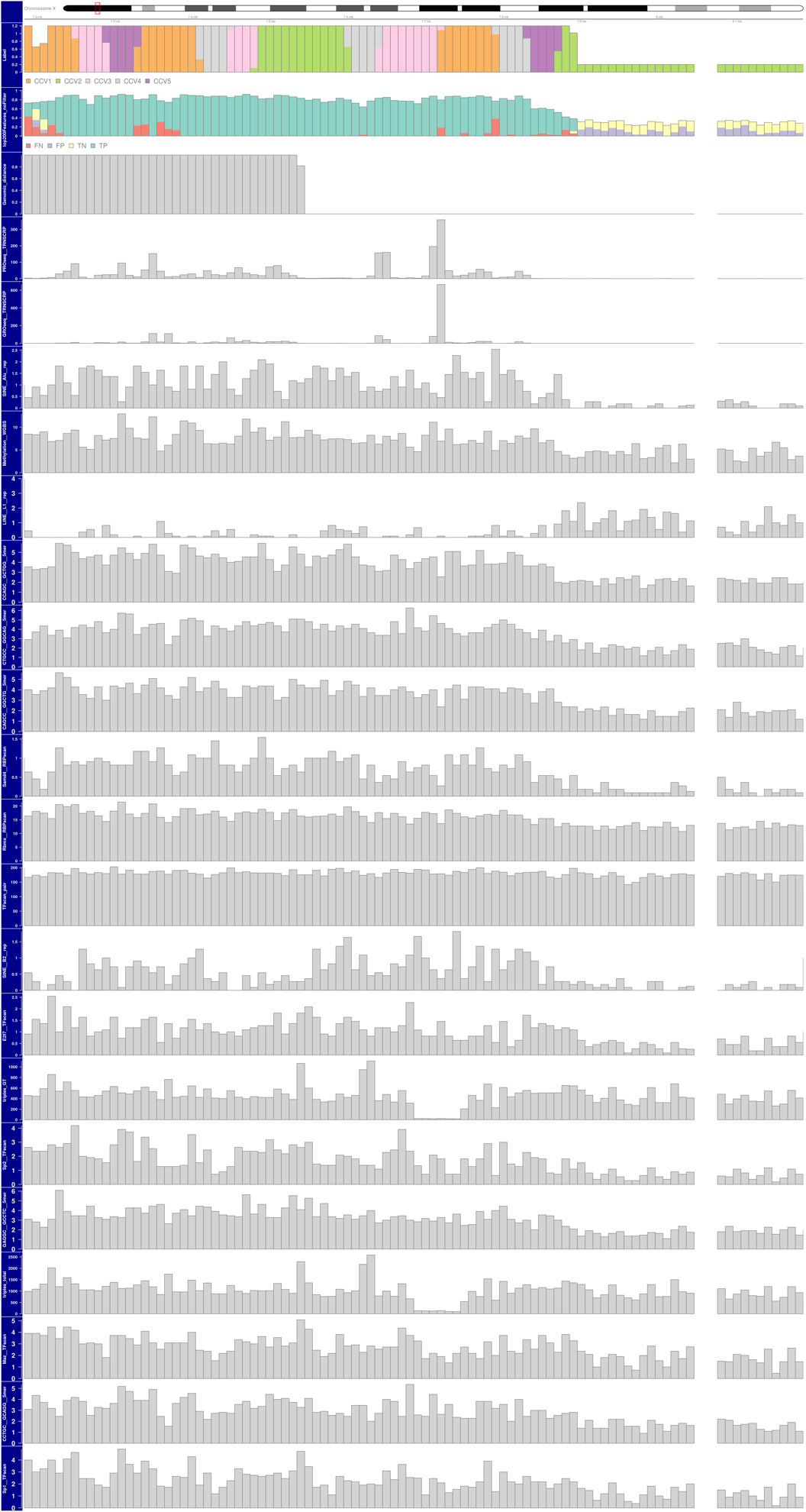


**Figure S1 (previous page).** Example of model predictions for Gm14820 together with predictive feature values. Top panel illustrates the labels for each of the tiles. Labels are averaged in sliding 10kb windows. Thus, a label of 0.6 would mean that the model predicted 60% of the tiles in that 10kb window to be bound. Since predictions are made within a cross-validation scheme, colors are used to indicate which fold of cross-validation included a particular window in its test set. Second panel illustrates the model predictions, colored as TP, FP, TN, and FN. Third panel illustrates the distance from lncRNA gene (in Mega basepair), truncated at one Mbp. Remaining panels profile important features identified by the model.


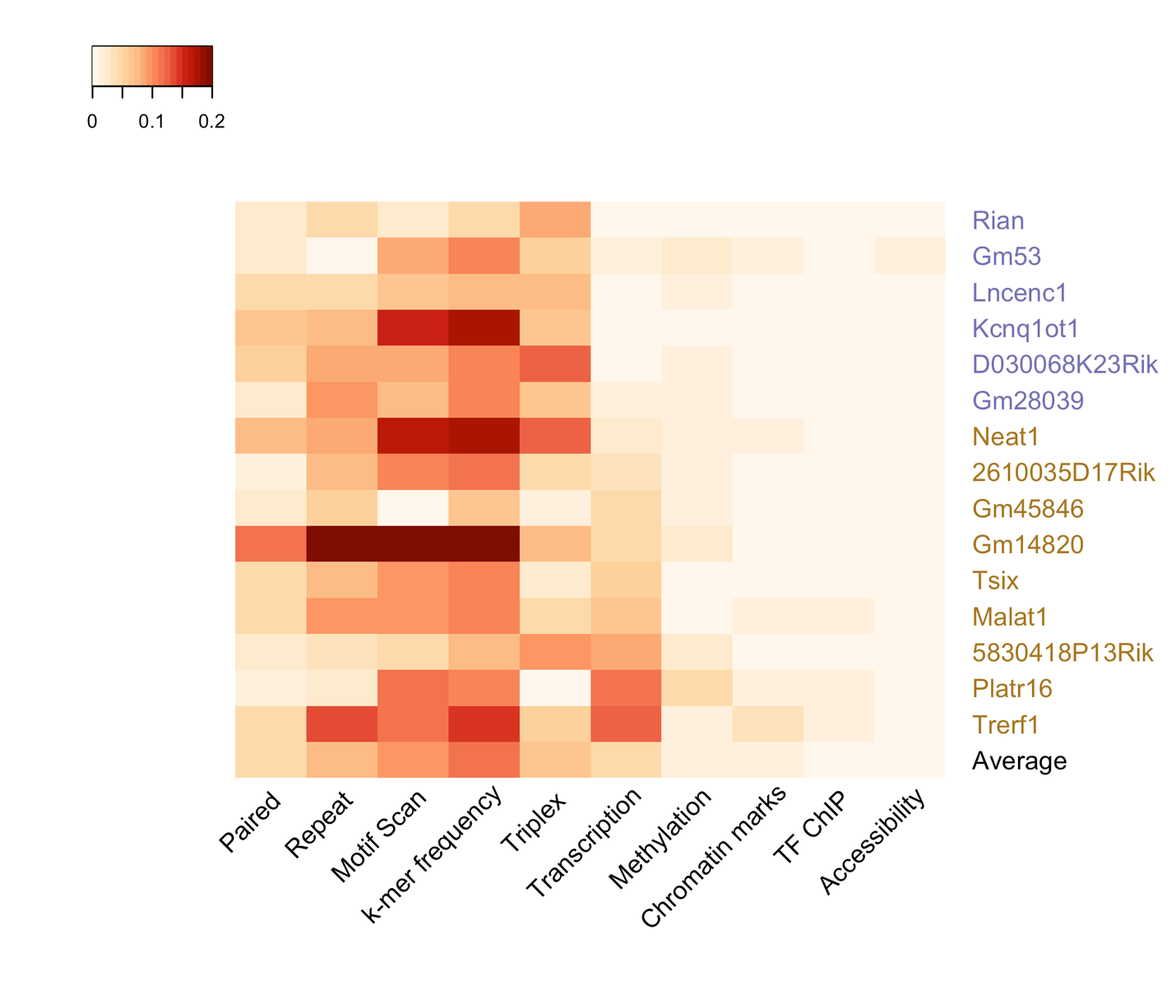


**Figure S2. Maximal Predictive Value (MxPV).** Heatmap shows MxPV of feature familes (columns) for all lncRNAs (rows). Row label colors show lncRNA group (TA and non-TA lncRNAs shown in gold and navy, respectively).


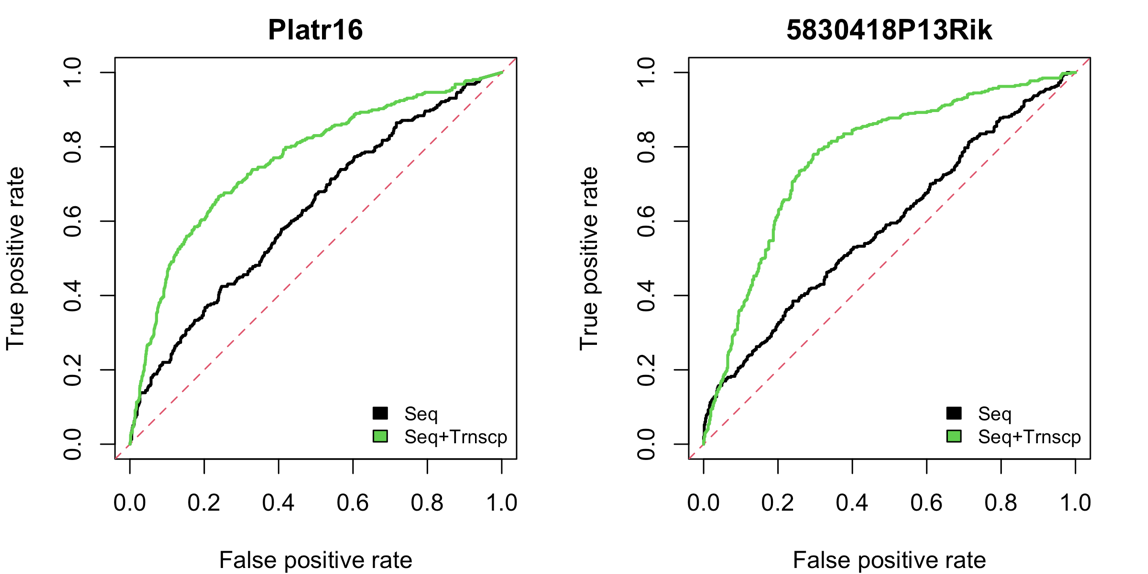


**Figure S3. Transcription feature family enhances model predictions.** Panels show test ROC curves representing the performance of models trained to predict Platr16- (left) and 5830418P13Rik- (right) chromatin interactions using sequence alone (black line), or together with transcription family of features (green line).


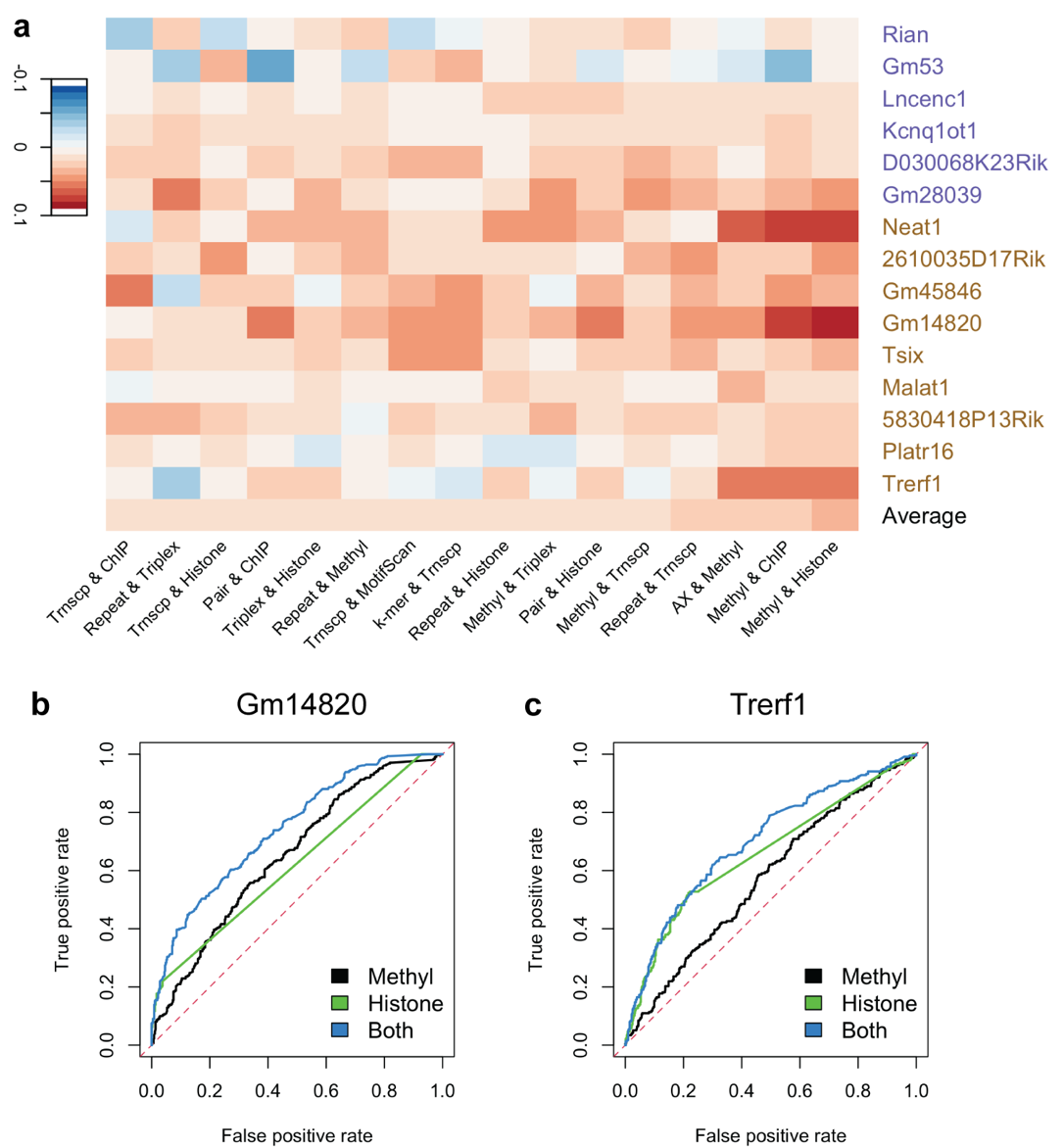


**Figure S4. Feature family complementarity**. (a) Heatmap showing complementarity score for 16 most complementary family pairs. Each row represents a lncRNA and each column corresponds to a pair of feature families. Row labeles are colored based on lncRNA type (TA lncRNAs are labeled in gold, whereas non-TA lncRNAs are labeled in navy). Values represent the difference between AUROC of a model trained with the pair of features, and the maximum AUROC achieved with either feature family alone. (b) and (c) show ROC curves reporting the performance of models trained to predict Gm14820 and Trerf1, respectively, using DNA Methylation, Histone marks, or both feature familes together. (Performance is from test data in cross-validation.)


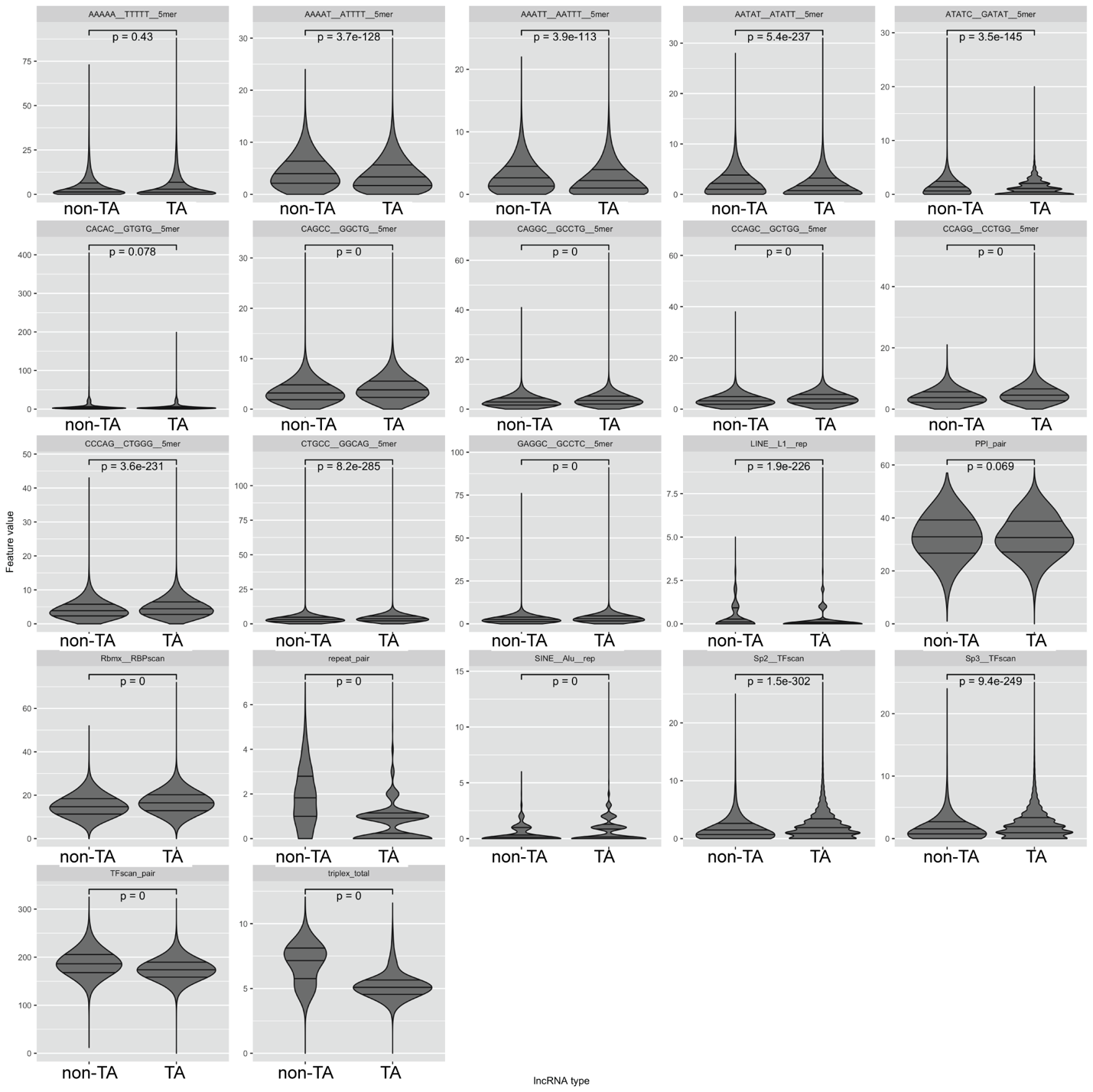


**Figure S5.** Violin plots showing distributions of selected sequence features in tiles bound by Transcription-associated (TA) lncRNAs and non-TA lncRNAs. P-values are shown as obtained by Kolmogorov-Smirnov test.


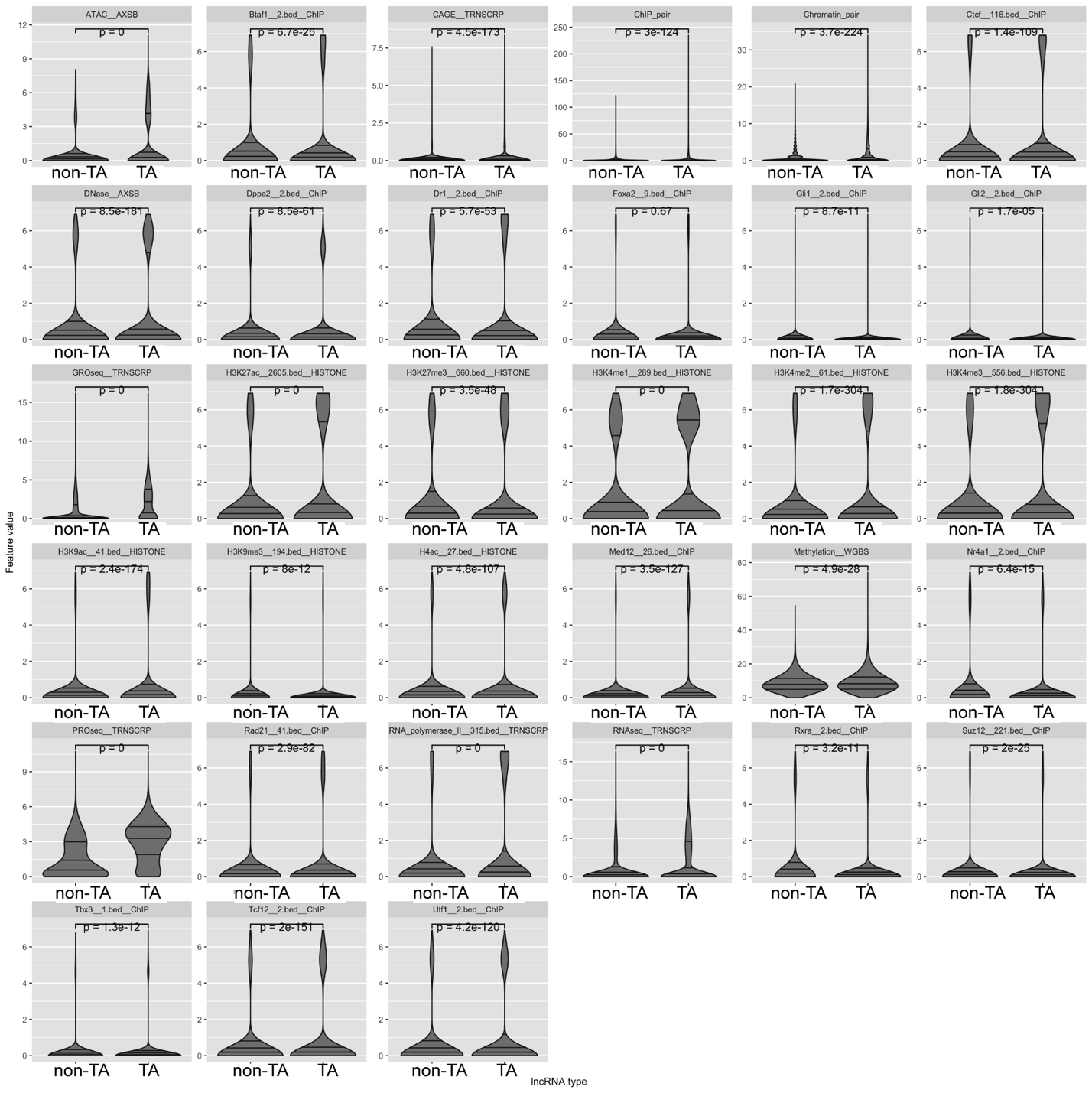


**Figure S6.** Violin plots showing distributions of selected context features in tiles bound by TA lncRNAs and non-TA lncRNAs. P-values are shown as obtained by Kolmogorov-Smirnov test.


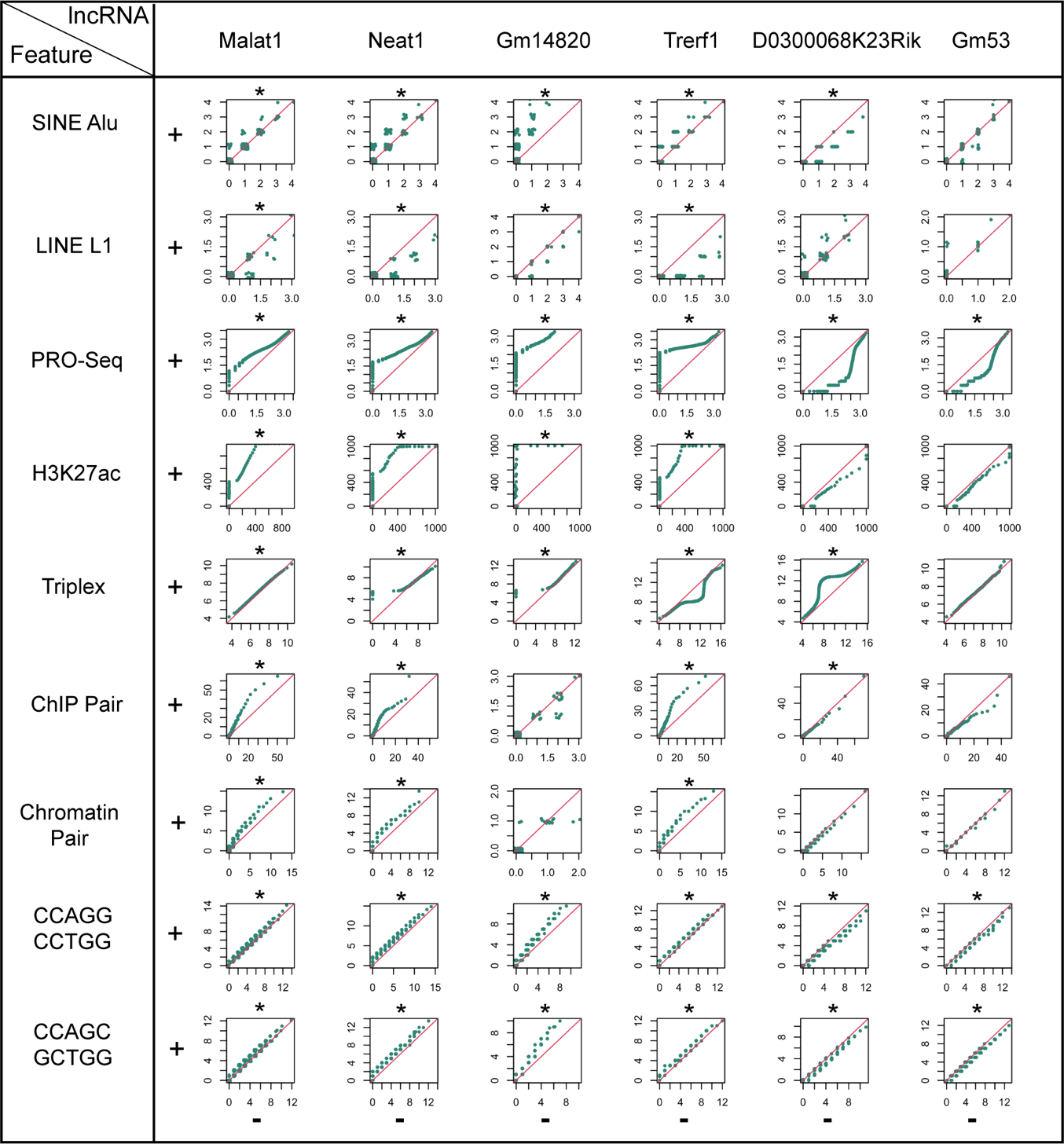


**Figure S7.** Visualization of features distinguishing bound vs non-bound tiles for selected lncRNAs. Quantile-Quantile plots show the distribution of feature values in bound DNA tiles (i.e., +) as compared to unbound ones (i.e., -). Each row corresponds to a feature and each column represents a lncRNA. For the purpose of effective visualization, we focused on a few lncRNAs. Selected lncRNAs include Malat1 and Neat1 due to their well-known biological importance; Gm14820, since the models performed exceptionally well in predicting its targets; Trerf1 as the only processed transcript overlapping 3’ UTR of a protein-coding gene with important features resembling those of Malat1 and Neat1; and finally, Gm53 and D0300068k23Rik because of their tendency to bind to regions with lower transcriptional activity. * indicates adjusted p-value < 0.01 obtained by Kolmogorov-Smirnov test.


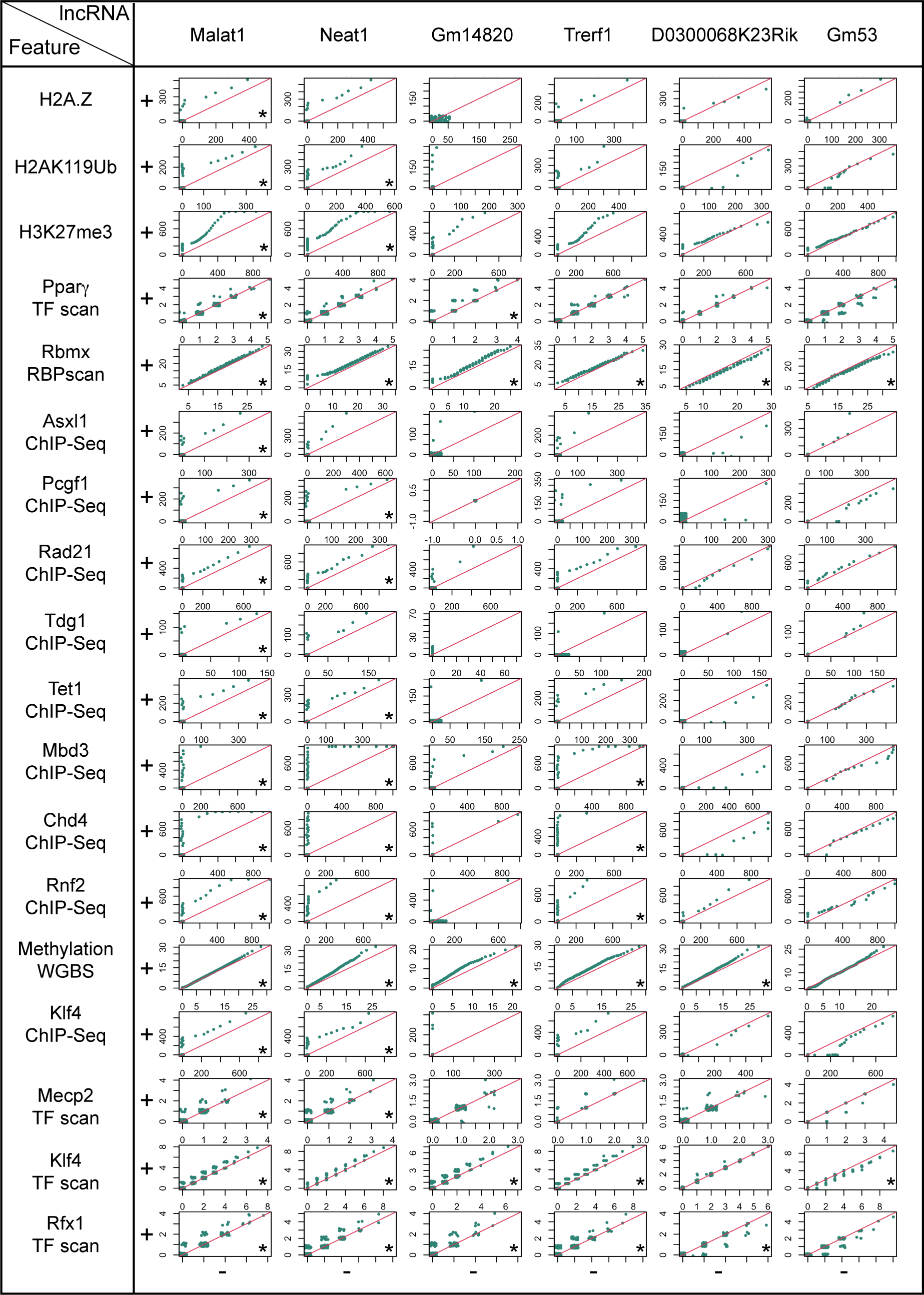


**Figure S8 (previous page).** Visualization of DNA Damage Response- and methylation- related features. Quantile-Quantile plots show the distribution of feature values in bound DNA tiles as compared to unbound ones. * indicates adjusted p-value < 0.01 obtained by Kolmogorov-Smirnov test.

**Supplementary Table 1**

Number of datapoints each representing a 1 kb DNA tile is listed in the positive (bound) and negative (unbound) sets for each of the studied lncRNAs.

| lncRNA | Negative | Positive |
| --- | --- | --- |
| 2410003L11Rik | 654 | 1111 |
| 2610035D17Rik | 3591 | 1092 |
| 5830418P13Rik | 2860 | 1993 |
| D030068K23Rik | 3225 | 2770 |
| E130307A14Rik | 6623 | 2775 |
| Firre | 3389 | 4843 |
| Gm11613 | 1155 | 1183 |
| Gm14820 | 1784 | 1511 |
| Gm28039 | 1895 | 1469 |
| Gm28052 | 1943 | 1021 |
| Gm28373 | 5593 | 1402 |
| Gm45846 | 3766 | 1406 |
| Gm53 | 907 | 1671 |
| Gnasas1 | 1945 | 2037 |
| Ino80dos | 4604 | 2694 |
| Kcnq1ot1 | 14446 | 5553 |
| Lncenc1 | 22047 | 7109 |
| Lncpint | 3298 | 1491 |
| Malat1 | 204566 | 110183 |
| Meg3 | 6988 | 2343 |
| Neat1 | 4389 | 2099 |
| Platr16 | 7387 | 1633 |
| Platr22 | 2421 | 1048 |
| Platr28 | 11260 | 3578 |
| Pvt1 | 44134 | 16362 |
| Rian | 4500 | 1565 |
| Trerf1 | 2632 | 1176 |
| Tsix | 14227 | 5014 |

**Supplementary Table 2**

| Dataset | Accession/Source |
| --- | --- |
| DNA accessibility | GSE113592 |
| DNA methylation | GSE130735 |
| GRO-Seq | GSE63974 |
| PRO-Seq | GSE130691 |
| RNA-Seq | GSE29184 |
| Chromatin marks | ChIP-Atlas |
| TF ChIP | ChIP-Atlas |

**Supplementary note 1**

Illustrated features in **supplementary figure S6** were selected among the ones (from the context features category) identified as important for the majority of lncRNAs, with distinctive patterns in the chosen lncRNAs. As shown in this figure, nascent transcription measured by PRO-Seq, and H3K27ac are clearly enriched in tiles interacting Malat1, Neat1, Gm14820, and Trerf1 but depleted in regions bound by Gm53 and D0300068k23Rik. Alu and Line-1 repeat elements show opposing patterns. Alu is enriched in regions bound by lncRNAs associated with active transcription and is depleted in regions bound by lncRNAs associated with transcriptional repression, while Line-1 follows an opposite trend.

Triplex-formation potential shows an interesting distinction between Trerf1 and D0300068k23Rik. This observation indicates that Trerf1 preferentially interacts with the chromatin around regions that are less likely to form a triplex with it, as opposed to D0300068k23Rik which interacts with regions of high triplex formation potential. This puzzling observation might be explained by the transcriptional status of the bound regions of each lncRNA.

Chromatin-pair feature, reflecting the chromatin context similarity between the DNA tile and the lncRNA gene, distinguishes the bound and unbound regions of Malat1, Neat1 and Trerf1, but is not informative about the chromatin interactions of Gm14820, Gm53, and D0300068k23Rik. ChIP-pair feature follows a similar pattern to that of chromatin-pair.

Finally, we compared the distribution of two 5-mers that were informative for the majority of lncRNAs. Both k-mers follow patterns similar to Alu repeats. This is consistent with the sequence similarities found between the 5-mers and Alu elements (1).

**Supplementary note 2: A closer look at methylation and DDR-related factors**

As illustrated in Figure 4, DNA Damage Repair (DDR) seems to be a common theme encompassing many of the identified factors predictive of lncRNA-chromatin interactions. In **supplementary figure S7**, we focus on differential enrichment of DDR-related features. As shown here, H2A.Z histone mark, known to be required for double strand break repair (2), is differentially present in tiles bound by Malat1, Neat1, and Trerf1. Among the histone modifications associated with DDR, H3K27me3 (3) and H2AK119Ub (4) are observed more frequently in DNA tiles bound by transcription-associated (TA) lncRNAs. Given the repressive nature of these chromatin marks, their association with lncRNAs that are more likely to bind areas of active transcription is puzzling.

Comparison of DNA binding affinity for Pparγ, a nuclear receptor TF involved in DDR (5), shows a similar pattern of enrichment in lncRNA-bound DNA tiles for Malat1, Neat1 and Gm14820. This pattern is reversed for Gm53.

Rbmx is found to be one of the most important sequence features for all considered lncRNAs. This RBP with significant roles in splicing was recently found to be essential in DDR. Rbmx is observed to localize to repetitive single stranded DNA and activate ATR in coordination with RPA (6).

Mecp2 binds to methylated cytosine both in CpG and non-CpG contexts and is involved in DDR. Defect and overexpression of Mecp2 are both associated with cognitive disorders, namely Rett syndrome and M2DS, respectively (7). Mecp2 is known to be part of miRNA processing machinery in cooperation with Rbmx (also known as hnRNPG) (8), and is known to interact with several miRNAs (9). Mecp2 is also known to be associated with alternative splicing (10) which is affected in Rett syndrome (11). Furthermore, Mecp2 interacts with multiple lncRNAs including Neat1 (12), Malat1, Meg3, Xist, Rian, and Kcnq1ot1 (13). We examined the sequence preference of this protein in the considered bound and unbound DNA regions. As shown in Figure S7 and consistent with previous reports of interaction between Mecp2, Malat1 and Neat1, we find this protein to generally have higher binding affinity for regions bound by Malat1 and Neat1.

Asxl1 is known to form a complex with BRCA associated protein 1 (BAP1) and mediate deubiquitination of H2AK119, a required step in DDR (14,15). Binding profile of this protein shows its relative enrichment in regions bound by Malat1, Neat1 and Trerf1.

Pcgf1 is a component of polycomb repressive group 1 (required for mono-ubiquitinylation of H2AK119) and is involved in targeting of this complex to CpG islands (16). ChIP experiments in mESC show relative binding enrichment of this protein in regions bound by Malat1, Neat1, and Trerf1, as well as a relative depletion in regions bound by Gm53.

Rad21 is a part of Cohesin complex and known as the double-strand break repair protein (17). As shown in Figure S7, this protein preferentially binds to regions interacting with Malat1, Neat1, Gm14820, and Trerf1.

Methylated cytosine has a high chance of mutation to thymine, creating a G.T mismatch. Particular enzymes are responsible for fixing this mutation through a process called Base Excision Repair (BER). Tdg is such an enzyme, and its function is known to be coupled with methylation as the newly replaced cytosine requires to be methylated (18). Tdg is also known to physically interact with Tet1 for actively demethylation and subsequent excision of the methylated cytosine (19). Tet2 has been observed to inhibit the action of Tet1-Tdg complex, while Rxra is shown to cooperate with the complex to achieve active demethylation of target sites (20). Tet1 is an enzyme responsible for active demethylation of cytosines (21). It oxidizes 5mC into 5-hydroxymethyl-cytosine (5-hmC), or other oxidized forms (including 5-formyl (fC), and 5-carboxylcytosine (caC)) each bound and read by a different set of proteins (22). Interestingly Tdg binds to fC and caC but not hmC. It has been reported that Rfx1, Klf4, Klf5 localize to methylated cytosines, whereas Kdm2b, Ino80, and Zbtb2 preferentially localize to unmethylated cytosines (22). Figure S7 shows the differential binding patterns of several of the mentioned proteins in lncRNA bound vs unbound DNA regions.

MBD3 is a methyl CpG binding protein and the only one known to preferentially bind to hydroxy-methylated DNA. Its abnormal expression and binding patterns are associated with Epilepsy (23). Mbd3 is known to be essential for methylation homeostasis (24), pluripotency, apoptosis (25), colocalize with Tet1, interact with HDAC1 and NuRD complex (26,27) . NuRD complex is known to be recruited to sites of DNA damage partly through its Cdh4 subunit (28) and facilitate DNA repair. MTA subunit of NuRD complex is also known to regulated by miRNAs (29). Given the differential binding (between lncRNA-bound and unbound tiles, shown in Figure S7) of a broad set of proteins related to dynamic methylation of cytosine, particularly Mbd3, Tet1, Chd4, as well as zinc finger proteins Klf4, and Klf5, we speculate that lncRNA-chromatin interactions are involved in this process in some form.

**References**

1. López E, Casasnovas C, Giménez J, Matilla-Dueñas A, Sánchez I, Volpini V. Characterization of Alu and recombination-associated motifs mediating a large homozygous SPG7 gene rearrangement causing hereditary spastic paraplegia. Neurogenetics [Internet]. 2015 Mar 18 [cited 2021 May 16];16(2):97–105. Available from: https://pubmed.ncbi.nlm.nih.gov/25398481/

2. Xu Y, Ayrapetov MK, Xu C, Gursoy-Yuzugullu O, Hu Y, Price BD. Histone H2A.Z Controls a Critical Chromatin Remodeling Step Required for DNA Double-Strand Break Repair. Mol Cell [Internet]. 2012 Dec 14 [cited 2021 May 16];48(5):723–33. Available from: https://pubmed.ncbi.nlm.nih.gov/23122415/

3. O’Hagan HM, Mohammad HP, Baylin SB. Double strand breaks can initiate gene silencing and SIRT1-dependent onset of DNA methylation in an exogenous promoter CpG island. PLoS Genet [Internet]. 2008 Aug [cited 2021 May 16];4(8). Available from: https://pubmed.ncbi.nlm.nih.gov/18704159/

4. Shanbhag NM, Rafalska-Metcalf IU, Balane-Bolivar C, Janicki SM, Greenberg RA. ATM-Dependent chromatin changes silence transcription in cis to dna double-strand breaks. Cell [Internet]. 2010 [cited 2021 May 16];141(6):970–81. Available from: https://pubmed.ncbi.nlm.nih.gov/20550933/

5. Li CG, Mahon C, Sweeney NM, Verschueren E, Kantamani V, Li D, et al. PPARγ Interaction with UBR5/ATMIN Promotes DNA Repair to Maintain Endothelial Homeostasis. Cell Rep [Internet]. 2019 Jan 29 [cited 2021 May 16];26(5):1333-1343.e7. Available from: https://pubmed.ncbi.nlm.nih.gov/30699358/

6. Zheng T, Zhou H, Li X, Peng D, Yang Y, Zeng Y, et al. RBMX is required for activation of ATR on repetitive DNAs to maintain genome stability. Cell Death Differ [Internet]. 2020 Nov 1 [cited 2021 May 15];27(11):3162–76. Available from: https://pubmed.ncbi.nlm.nih.gov/32494026/

7. Chahrour M, Sung YJ, Shaw C, Zhou X, Wong STC, Qin J, et al. MeCP2, a key contributor to neurological disease, activates and represses transcription. Science (80- ) [Internet]. 2008 May 30 [cited 2021 May 16];320(5880):1224–9. Available from: https://pubmed.ncbi.nlm.nih.gov/18511691/

8. Tsujimura K, Irie K, Nakashima H, Egashira Y, Fukao Y, Fujiwara M, et al. MiR-199a Links MeCP2 with mTOR Signaling and Its Dysregulation Leads to Rett Syndrome Phenotypes. Cell Rep [Internet]. 2015 Sep 22 [cited 2021 May 15];12(11):1887–901. Available from: https://pubmed.ncbi.nlm.nih.gov/26344767/

9. Khan AW, Ziemann M, Rafehi H, Maxwell S, Ciccotosto GD, El-Osta A. MeCP2 interacts with chromosomal microRNAs in brain. Epigenetics [Internet]. 2017 Dec 2 [cited 2021 May 16];12(12):1028–37. Available from: https://pubmed.ncbi.nlm.nih.gov/29412786/

10. Wong JJL, Gao D, Nguyen T V., Kwok CT, Van Geldermalsen M, Middleton R, et al. Intron retention is regulated by altered MeCP2-mediated splicing factor recruitment. Nat Commun [Internet]. 2017 May 8 [cited 2021 May 16];8. Available from: https://pubmed.ncbi.nlm.nih.gov/28480880/

11. Li R, Dong Q, Yuan X, Zeng X, Gao Y, Chiao C, et al. Misregulation of Alternative Splicing in a Mouse Model of Rett Syndrome. PLoS Genet [Internet]. 2016 Jun 1 [cited 2021 May 16];12(6). Available from: https://pubmed.ncbi.nlm.nih.gov/27352031/

12. Cheng C, Spengler RM, Keiser MS, Monteys AM, Rieders JM, Ramachandran S, et al. The long non-coding RNA NEAT1 is elevated in polyglutamine repeat expansion diseases and protects from disease gene-dependent toxicities. Hum Mol Genet [Internet]. 2018 Dec 15 [cited 2021 May 16];27(24):4303–14. Available from: https://pubmed.ncbi.nlm.nih.gov/30239724/

13. Maxwell SS, Pelka GJ, Tam PP, El-Osta A. Chromatin context and ncRNA highlight targets of MeCP2 in brain. RNA Biol [Internet]. 2013 [cited 2021 May 16];10(11):1741–57. Available from: https://pubmed.ncbi.nlm.nih.gov/24270455/

14. Sahtoe DD, Van Dijk WJ, Ekkebus R, Ovaa H, Sixma TK. BAP1/ASXL1 recruitment and activation for H2A deubiquitination. Nat Commun [Internet]. 2016 Jan 7 [cited 2021 May 16];7. Available from: https://pubmed.ncbi.nlm.nih.gov/26739236/

15. Uckelmann M, Sixma TK. Histone ubiquitination in the DNA damage response [Internet]. Vol. 56, DNA Repair. Elsevier B.V.; 2017 [cited 2021 May 16]. p. 92–101. Available from: https://pubmed.ncbi.nlm.nih.gov/28624371/

16. Blackledge NP, Farcas AM, Kondo T, King HW, McGouran JF, Hanssen LLP, et al. Variant PRC1 complex-dependent H2A ubiquitylation drives PRC2 recruitment and polycomb domain formation. Cell [Internet]. 2014 Jun 5 [cited 2021 May 16];157(6):1445–59. Available from: https://pubmed.ncbi.nlm.nih.gov/24856970/

17. Cheng H, Zhang N, Pati D. Cohesin subunit RAD21: From biology to disease [Internet]. Vol. 758, Gene. Elsevier B.V.; 2020 [cited 2021 May 16]. Available from: https://pubmed.ncbi.nlm.nih.gov/32687945/

18. Walsh CP, Xu GL. Cytosine methylation and DNA repair. In: Current Topics in Microbiology and Immunology [Internet]. Springer Verlag; 2006 [cited 2021 May 16]. p. 283–315. Available from: https://pubmed.ncbi.nlm.nih.gov/16570853/

19. Weber AR, Krawczyk C, Robertson AB, Kusnierczyk A, Vågbø CB, Schuermann D, et al. Biochemical reconstitution of TET1-TDG-BER-dependent active DNA demethylation reveals a highly coordinated mechanism. Nat Commun [Internet]. 2016 Mar 2 [cited 2021 May 16];7. Available from: https://pubmed.ncbi.nlm.nih.gov/26932196/

20. Hassan HM, Kolendowski B, Isovic M, Bose K, Dranse HJ, Sampaio A V., et al. Regulation of Active DNA Demethylation through RAR-Mediated Recruitment of a TET/TDG Complex. Cell Rep [Internet]. 2017 May 23 [cited 2021 May 16];19(8):1685–97. Available from: https://pubmed.ncbi.nlm.nih.gov/28538185/

21. Tahiliani M, Koh KP, Shen Y, Pastor WA, Bandukwala H, Brudno Y, et al. Conversion of 5-methylcytosine to 5-hydroxymethylcytosine in mammalian DNA by MLL partner TET1. Science (80- ) [Internet]. 2009 May 15 [cited 2021 May 16];324(5929):930–5. Available from: https://pubmed.ncbi.nlm.nih.gov/19372391/

22. Spruijt CG, Gnerlich F, Smits AH, Pfaffeneder T, Jansen PWTC, Bauer C, et al. Dynamic readers for 5-(Hydroxy)methylcytosine and its oxidized derivatives. Cell [Internet]. 2013 Feb 28 [cited 2021 May 16];152(5):1146–59. Available from: https://pubmed.ncbi.nlm.nih.gov/23434322/

23. Bednarczyk J, Dȩbski KJ, Bot AM, Lukasiuk K. MBD3 expression and DNA binding patterns are altered in a rat model of temporal lobe epilepsy. Sci Rep [Internet]. 2016 Sep 21 [cited 2021 May 16];6. Available from: https://pubmed.ncbi.nlm.nih.gov/27650712/

24. Cui Y, Irudayaraj J. Dissecting the behavior and function of MBD3 in DNA methylation homeostasis by single-molecule spectroscopy and microscopy. Nucleic Acids Res [Internet]. 2015 Mar 31 [cited 2021 May 16];43(6):3046–55. Available from: https://pubmed.ncbi.nlm.nih.gov/25753672/

25. Dai Y, Li J, Li M, Liu Z, Liu J, An L, et al. Methyl-CpG-binding domain 3 (Mbd3) is an important regulator for apoptosis in mouse embryonic stem cells. Am J Transl Res. 2020;12(12):8147–61.

26. Du Q, Luu PL, Stirzaker C, Clark SJ. Methyl-CpG-binding domain proteins: Readers of the epigenome [Internet]. Vol. 7, Epigenomics. Future Medicine Ltd.; 2015 [cited 2021 May 16]. p. 1051–73. Available from: https://pubmed.ncbi.nlm.nih.gov/25927341/

27. Yildirim O, Li R, Hung JH, Chen PB, Dong X, Ee LS, et al. Mbd3/NURD complex regulates expression of 5-hydroxymethylcytosine marked genes in embryonic stem cells. Cell [Internet]. 2011 Dec 23 [cited 2021 May 16];147(7):1498–510. Available from: https://pubmed.ncbi.nlm.nih.gov/22196727/

28. Chou DM, Adamson B, Dephoure NE, Tan X, Nottke AC, Hurov KE, et al. A chromatin localization screen reveals poly (ADP ribose)-regulated recruitment of the repressive polycomb and NuRD complexes to sites of DNA damage. Proc Natl Acad Sci U S A [Internet]. 2010 Oct 26 [cited 2021 May 16];107(43):18475–80. Available from: https://pubmed.ncbi.nlm.nih.gov/20937877/

29. Zhang Y, Wang XF. Post-transcriptional regulation of MTA family by microRNAs in the context of cancer. Cancer Metastasis Rev [Internet]. 2014 Nov 26 [cited 2021 May 16];33(4):1011–6. Available from: https://pubmed.ncbi.nlm.nih.gov/25332146/
